# Smart bistable coordination complexes

**DOI:** 10.1002/smo.20230028

**Published:** 2024-05-23

**Authors:** Xiong Xiao, Zong‐Ju Chen, Russell J. Varley, Cheng‐Hui Li

**Affiliations:** ^1^ State Key Laboratory of Coordination Chemistry School of Chemistry and Chemical Engineering Collaborative Innovation Center of Advanced Microstructures Nanjing University Nanjing China; ^2^ Carbon Nexus at the Institute for Frontier Materials Deakin University Waurn Ponds Victoria Australia

**Keywords:** bistable, coordination chemistry, dynamic bonds, smart molecules, stimuli responsive

## Abstract

Smart molecules have attracted increasing attention due to their transformative role in creating the next generation of smart structures and devices. Smart bistable coordination complexes are a class of functional complexes which have two stable states that can be reversibly switched in response to external stimuli. Such bistable molecules play a vital role in various applications, such as sensors, data storage, spintronics, smart windows, optical switches, information encryption and decryption, displays, actuators, etc. Herein, the recent research studies into the development of these smart bistable metal coordination complexes are reviewed. According to the different external stimuli, these smart bistable coordination systems have been classified and summarized, including light‐responsive systems, thermally‐responsive systems, electrically‐responsive systems, mechanically‐responsive systems, and some other cases. These systems are further subdivided according to the changes in signals (e.g., color, fluorescence, spin state, crystalline phase) under external stimuli. The design principles of each type of smart bistable metal complexes as well as their broad and innovative applications are comprehensively described. Finally, the challenges and opportunities in this field are briefly analyzed and discussed.

## Graphical abstract

Smart bistable complexes play a vital role in various applications such as sensors, data storage, spintronics, smart windows, optical switches, information encryption and decryption, displays, actuators, and so on. In this review, we summarize smart bistable complexes in terms of external stimulus approach and discuss their design guidelines, properties, and applications.

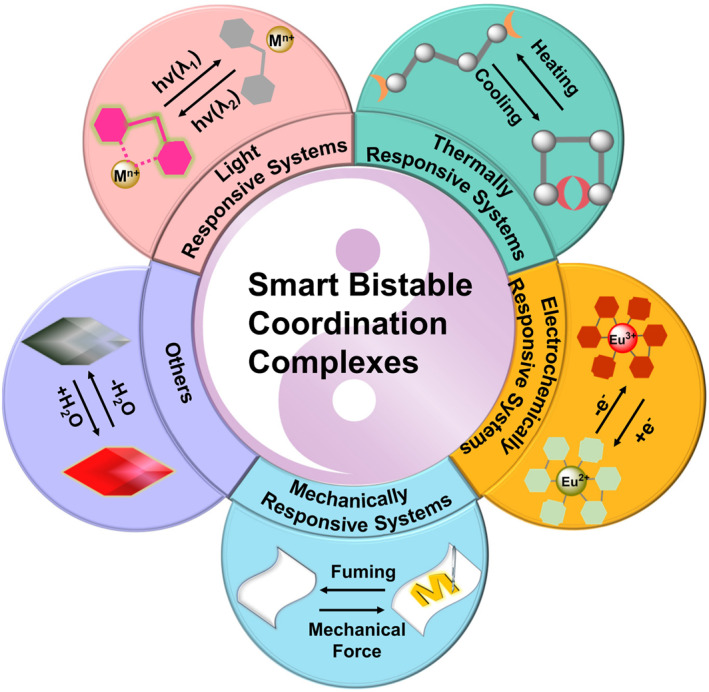

## 1 INTRODUCTION

Smart molecules are molecules or molecule‐based functional systems that show a response to external stimuli by means of a change of signal emission or action, such as light, heat, electric or magnetic fields, sound, stress, pH, moisture, guest molecules, etc.^[1]^ Controlling these responses is leading to innovative applications such as molecular machines that work as tiny robots for disease detection or drug delivery of microprocessors to specific sites.^[2]^ Another example is organic dyes that can change their absorption or fluorescent/phosphorescent under external stimuli and used in developing mechano‐, piezo‐, and thermo‐fluorochromes materials in the optical recording of information, sensors, security items, memory elements, and organic light‐emitting diodes (OLED) technologies.^[3]^ For catalysis, smart molecules can act as reversible photo‐switches for enantiospecific transformations and reversible photo‐superstructures, digital photo‐programming, and tunable circularly polarized luminescence (CPL) with a high dissymmetry factor.^[4]^ It is also proposed that smart molecules can mimic advanced logic operations for the next generation of computers and devices.^[5]^

Among the various smart molecules, bistable molecules are particularly interesting as their physical properties can be reversibly switched between two different stable states^[6]^ by external stimuli, and thus are very useful for applications where switching functions are required.^[7]^ Moreover, bistability is commonly observed in nature and daily life, for example, the flytrap has two stable states with an open‐convex shape and a closed‐concave shape, thus can lock insects in its lobes.^[8]^ Therefore, the investigation of bistable materials is helpful for understanding natural phenomena. So far, numerous bistable phenomena have been observed in various fields, ranging from ferromagnetism^[9]^ and ferroelectrics^[10]^ to spin‐crossover,^[11]^ electrochromism^[12]^ and photochromic.^[13]^ Similarly, a diverse range of bistable molecules have been developed, including azobenzene,^[14]^ spiropyrans,^[15]^ and diarylethene.^[16]^ These bistable materials have found significant applications in various technological areas due to their unique ability to switch their properties in response to external stimuli.^[17]^ For instance, they can be utilized in information processing, data storage, and advanced display technologies. These bistable materials possess remarkable switching capabilities that can lead to innovative technological advancements in various fields.

Recently, the focus of materials research has shifted from purely inorganic or organic materials to organic‐inorganic hybrids for the development of increasingly complex and powerful smart materials and devices.^[18]^ In this scenario, metal complexes offer unique advantages as bistable molecules due to the interplay between the inorganic metal and the organic ligand. In view of the vast variety of coordination motifs, as well as the diversified physical and chemical properties that originated from the introduction of functional metal ions and/or ligands, a plethora of metal‐containing bistable molecules with advanced functionalities can be prepared. Moreover, compared with other dynamic chemical bonds, metal‐coordination bonds have the advantage that their bond strength and dissociation rate can be tuned to a greater extent. However, no systematic review has been conducted to date which focuses on smart bistable coordination complexes.

**FIGURE 1:**
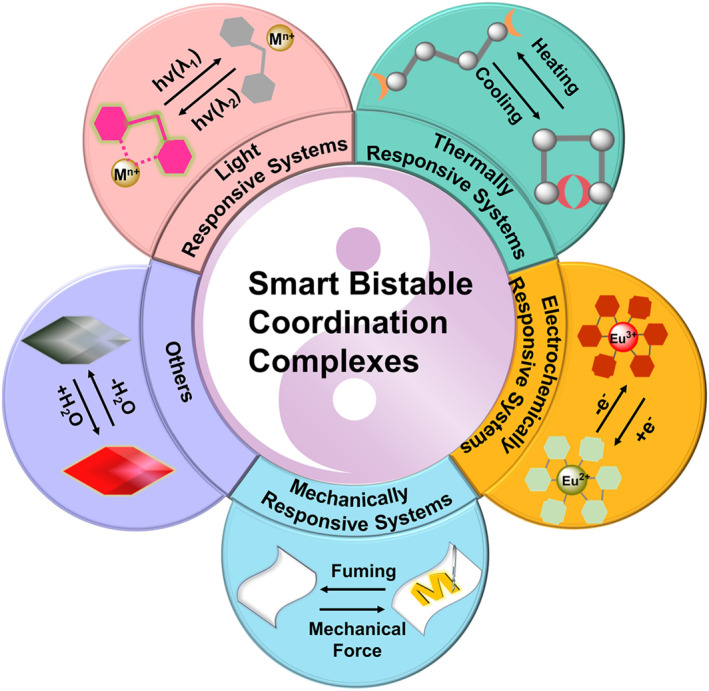
Overview of smart bistable coordination complexes classified by responding to different external stimuli.

Herein, the advances in the research of smart bistable coordination complexes are summarized by selecting some pivotal examples. Since nanomaterial,^[19]^ Metal–Organic Frameworks,^[20]^ polymer‐based advanced functional systems,[^8^, ^21^] switchable metallacycles and metallacages^[22]^ have been well reviewed, they will not be discussed in this paper. This review will focus on smart bistable coordination complexes comprised of small molecules and also provide a few examples of polynuclear metal complexes or two‐dimensional coordination polymers (CPs) constructed from functional small molecules. Generally, these small molecules function predominantly as ligands, facilitating the formation of complexes with metal ions. They possess structures capable of reversible transitions between two homogeneous states or feature functional groups proficient in chelating with metal ions or exerting electronic influence on metal centers. These smart bistable coordination complexes exhibit reversible alterations in color, fluorescence, magnetic properties, crystalline phases, and more when exposed to physical stimuli (such as light, temperature, electricity, and mechanical forces) and chemical analytes (including pH, gas molecules, and ions) (Figure 1). Considering that bistable smart materials can undergo single or multiple property changes in response to diverse external stimuli, this review is divided into several sections from the perspective of external stimuli and further subdivided according to the composition or properties of the smart material. For clarity, the categories used here are not completely independent of each other. For instance, a structure can undergo transformation under light stimulation, but its structural restoration may require other stimuli such as heat. Alternatively, the structural transformation could be achieved with multiple external stimuli.

This review is structured as follows. Section 2 briefly presents a general overview of the origin of bistability in coordination complexes and outlines the design strategy of smart bistable complexes; Section 3 reviews some representative examples of smart bistable complexes that convert information into visual signals such as absorbance and fluorescence in response to light stimulation, and demonstrates their advantages in areas such as chemical sensors, anti‐counterfeiting technology, and security printing. Section 4 summarizes various examples of smart coordination‐based thermally responsive molecules that respond to temperature changes with conformational transitions and crystalline phase changes. Section 5 provides a summary of various electrochemically responsive bistable complexes that exhibit electrochromic (EC), electro‐fluorescent, and redox changes upon electrical stimulation. Section 6 briefly describes several examples of reversible transformation of complexes induced by mechanical force stimuli including pressure, tension and grinding. Section 7 shows examples of bistable complexes under stimuli such as chemical analytes (including pH, water, organic solvents) demonstrating their advantages in biosensing and bioimaging applications. Also, examples of multi‐stimulation or multi‐processing are briefly presented to illustrate their enormous potential for multi‐stimulation response applications, which opens new avenues for the design of novel advanced materials.

## 2 ORIGIN OF BISTABILITY AND GENERAL DESIGN STRATEGY OF BISTABLE COORDINATION COMPLEXES

**FIGURE 2:**
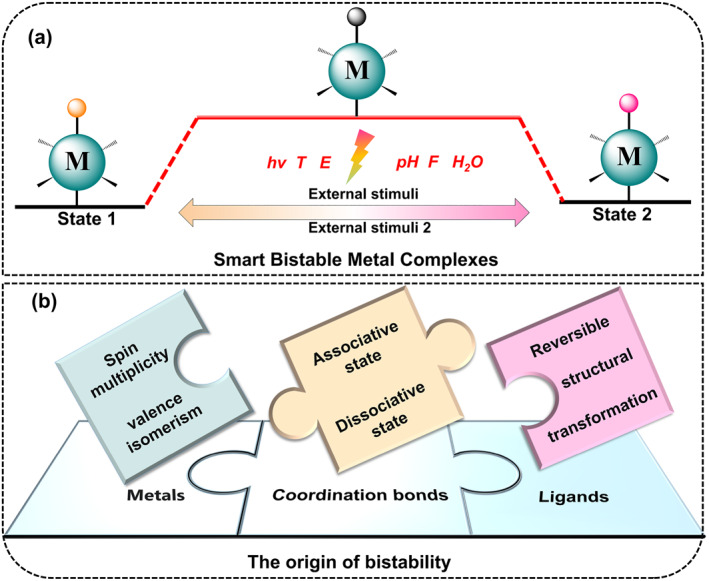
(a) Smart bistable coordination complexes achieve reversible transitions between different stable states in response to external stimuli, such as light (*hν*), temperature (*T*), electric fields (*E*), pH, mechanical force (*F*), and water (H_2_O). (b) Critical factors within coordination complexes that control bistability.

Coordination bonds, formed between a ligand providing lone pairs of electrons and a central atom or ion with a vacant orbital that accepts the lone pair electrons, are uniquely fascinating. Its strength lies between covalent and van der Waals interactions.^[23]^ Coordination bonds typically undergo dissociation or recombination more rapidly than typical covalent bonds in organic compounds and display comparatively better dynamics than covalent bonds.^[24]^ Specifically, most metal complexes of 3d transition metals are labile, which facilitates rapid ligand exchange reactions.[^17^, ^25^] The reversibility of coordination bonds creates a fascinating dynamic behavior of metal‐containing structures. Having sufficient structural flexibility and tunability, they do not exist as a stable single species but always undergo structural transitions which can be readily controlled.^[21c]^ Moreover, the ligand or metal centers of the complexes possess properties such as luminescence, dielectricity, and magnetism, which lead to multifunctional bistable complexes. As shown in Figure 2a, metal complexes with bistability exist in two thermodynamically stable forms (state **1** and state **2**). These two stable forms undergo transitions in response to diverse external stimuli such as light (*hν*), temperature (*T*), electric fields (*E*), pH, mechanical force (*F*), and water (H_2_O), accompanied by reversible transformations in physical properties that serve as signals for recording and reading. For these reasons, driven by new technologies and market trends, it is of great significance to analyze the property‐structure relationships of these compounds and explore the next generation of advanced functional materials.

The bistability of coordination complexes comes from three aspects (Figure 2b). First, some metal ions have spin multiplicity that can be shifted by external stimuli, leading to bistability. For example, the first‐row transition metal ions with d^4^–d^7^ electronic configurations can be switched between high spin (HS) state and low spin (LS) state by external stimuli, leading to spin crossover (SCO) bistable complexes. On the other hand, some metal complexes exhibit valence tautomeric behavior usually caused by metal‐metal or metal‐ligand (M‐L) electron transfer. Under external stimuli, metal complexes with valence isomerism exhibit interconversion between redox isomers, resulting in bistability. Second, some ligands show reversible structure transitions between two iso‐structures upon external stimuli. Coordination complexes containing such bistable ligands will also show bistability. For example, metal complexes with the 5,6‐dithienyl‐1,10‐phenanthroline ligand can switch between open conformation and closed conformation with significant change in color due to the photo‐switching behavior of dithienylethene (DTE) unit. Finally, due to the breaking and reformation of coordination bonds, complexes can switch from associated to dissociated states with different coordination numbers and conformations, also leading to bistability.

To construct efficient bistable coordination systems, several issues need to be addressed.^[26]^ First of all, the structural design of the bistable coordination system must select a suitable ligand and metal center, that is, the structural transformation of the ligand in response to an external stimulus may induce a change in the coordination properties of the metal center (dissociation of a coordination bond, change in the number of ligands, etc.), and the metal component should respond to the change and initiate the desired function. Secondly, the synthesized adducts should retain the desired properties, such that the addition of the metal center may lead to a delay or acceleration of the stimulus response but not an extinction. Third, such a coordination system is dynamically tunable, so that after a shift in response to an external stimulus, it can be restored to its initial state under a given condition and be repeated several times. Last but not least, as the key aspect to distinguish it from other stimulus‐responsive systems, both states before and after external stimulation have a certain stability which can be observed or detected prior to returning to the initial state after the withdrawal of the external stimulation.

As previously mentioned, bistability stems from compounds that undergo reversible and controllable transitions among two distinct molecular states when subjected to a particular external stimulus, such as light, temperature, or an electric field.^[6b]^ When we refer to controllable, we imply that the applied stimulus is considered as the primary driving force behind the state transition. In other words, at the beginning, the energy of both states is at a lower level than the threshold of the intermediate transition state.^[20b]^ When exposed to an external stimulus, the molecule acquires energy, enabling it to overcome the “energy barrier” and transition from one state to another, and vice versa (Figure 2a). This transition is bistable and removal of the stimulus does not immediately return the compound to its previous state, but requires actively reapplying the stimulus.^[7b]^ Therefore, irreversible processes and activation energies that are not sufficiently substantial for precise control or states that are too similar to be distinguished from each other do not meet the criteria for constructing bistable systems.^[20b]^ For example, some remarkably stable complexes exhibit small equilibrium constants in exchange reactions with other possible structures and tend to exist only as stable single species.^[18d,18e]^ As a result, this leads to prolonged response times to external stimuli, complexity in capturing signal changes and characterizing structural transformations. Additionally, structural transformations may be compelled to occur under extreme conditions such as high pressure, elevated temperature, and other potentially hazardous stimulus methods. On the contrary, some labile complexes lose their original properties under slight perturbations from the external environment or even undergo ligand exchange processes in solution, making it difficult to realize the ability to switch under a given condition of external stimuli.^[23]^ Hence, complexes exhibiting these characteristics will be excluded from the category of smart bistable molecules. According to the literature reported so far, the approaches to develop smart bistable metal complexes focus on two main aspects. One is the transformation of the ligand structure, such as the breaking, generation or rearrangement of chemical bonds or *cis*‐*trans* isomerism or conformational changes in response to an external stimulus.[^18^, ^27^] The second is the change of the metal center, including the acquisition or loss of electrons and modifications in the coordination mode and valence state.^[28]^ Undoubtedly, there is a wealth of sources for these ligands and metal centers. For instance, through the chelation of bistable photoswitch molecules to transition metal ions, efficient systems with photocontrolled charge and energy transfer are produced.[^6^, ^15^] Numerous redox‐driven metal complexes and organometallic compounds undergo structural reconfiguration upon exposure to electrochemical stimuli, thereby enabling the development of highly efficient electrically responsive bistable systems. The mechanism and kinetics of these processes, particularly metal‐to‐ligand charge transfer (MLCT), can be altered within the electronically excited state of these complexes.^[17c]^ The reorganization of molecular switches induces alterations in the geometry of the composite system, which enhances the interaction between the donor and acceptor components and favors the donor‐to‐acceptor CT. Furthermore, the emission characteristics of certain transition metal complexes demonstrate mechanical sensitivity in the presence of intermolecular interactions, including metal‐metal contacts and π‐π stacking interactions. This reversibility is achievable through processes such as recrystallization or heating.^[17c]^ In the case of complexes responsive to solvents, bistability frequently relies on the polycrystallization of the compound or the formation of solvates. Notably, these processes typically involve ligand exchange, and also presence of metal‐metal, solvent‐metal, π‐π stacking, and hydrogen bonding interactions. Bistable spin‐crossover compounds (SCOs) have the ability to transition between high‐spin (HS) and low‐spin (LS) ground states in response to external stimuli.^[11]^ This dynamic behavior is contingent upon the ligand field strength within the coordination environment, specifically when the splitting energy (Δ) approximates the pairing energy (*P*). External perturbations, such as temperature variations, light exposure, and applied pressure, can surmount the energy barriers between HS and LS states.^[17a]^ To conclude, bistable coordination complexes span across various research domains, incorporating a range of ligands and central metals considered as candidates for their construction. Detailed descriptions of these aspects will be provided in subsequent sections.

## 3 LIGHT RESPONSIVE SYSTEMS

Light is considered to be a near‐perfect external trigger for molecular systems, benefiting from the advantages that its stimulation is gentle and non‐polluting, has a wide window of spectral wavelengths and conveys information in the form of wavelengths and intensities, can be precisely controlled in space and time, and produces energy that can be reversibly converted into molecular motion, polarity, or flexibility changes.^[29]^ Dynamic metal coordination‐based photo‐responsive materials undergo reversible ligand bond dissociation or chromophore conversion accompanied by photo‐controlled CT and energy transfer under light stimulation, resulting in photochromic and magnetic transformations and other interesting properties.[^6^, ^26^, ^30^] Therefore, in recent years, M‐L coordination photo‐responsive materials are vital in many fields, such as smart windows,^[31]^ photo‐switches,^[32]^ photo‐switchable receptors,^[33]^ and other light‐responsive advanced materials.^[34]^

### 3.1 Photochromic bistable coordination complexes

Photochromism is a phenomenon whereby certain compounds can undergo reversible changes in structure that exhibit different optical properties upon light irradiation.[^29^, ^35^] Photochromic systems based on coordination complexes are very interesting due to the fact that organic photochromic systems prefer triplet states in the presence of spin‐orbit couplings from metal centers (especially transition metals) and allow the use of lower‐energy excitations or less destructive long‐wave excitations.[^29^, ^36^] The resulting long‐lived triplet excited states offer more possibilities for energy or electron transfer processes.^[37]^ Some exemplary photochromic agents include spiropyrans (SPs), diacetylenes (diaryl ethylenes (DAEs)), and dihydropyrenes (DHPs), which display an excellent reversible light response, discrete conversion, and quite high stability.^[6]^

**FIGURE 3:**
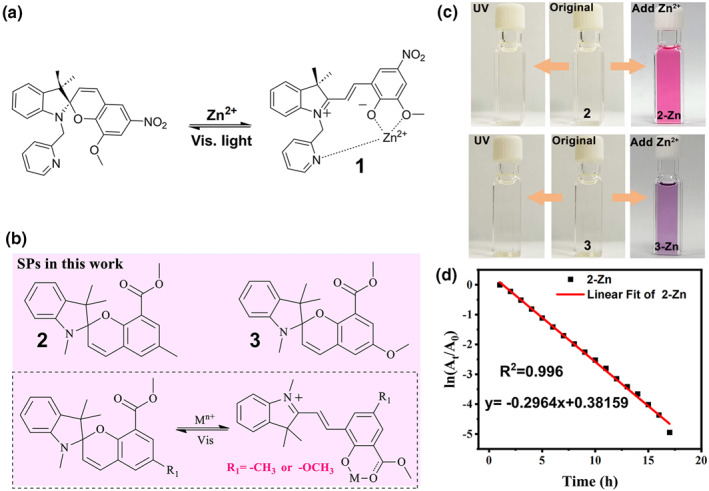
(a) Reversible interconversion between the SP state and complex **1**. Reproduced with permission: copyright 2010, Elsevier.^[40]^ (b) Structures and photochromic process of complexes **2** and **3**; (c) Photographs of the original SPs solution and after UV irradiation as well as photographs of solutions of the complexes **2** and **3**; (d) Fading kinetic curve of complexes **2**, Reproduced with permission: copyright 2023, Elsevier.^[41]^

Since their discovery in 1952, spiropyrans have contributed greatly to the development of photochromic materials.^[38]^ Combining spiropyran derivatives and their analogs (such as spirooxazine and spirothiopyran) with metal centers has imparted a variety of new properties such as redox, optical and magnetic properties of metal complexes.^[30a]^ The phenolic oxygen atoms in the ring‐opening merocyanine (MC) species of spiropyran have strong coordinating ability with metal ions and have chromogenic and fluorescent responses upon complexation with metal ions.^[39]^ For instance, Natali et al. grafted methoxy groups at the 8′ position of spiropyran and modified methylpyridyl group on indole nitrogen to form a new metal ion receptor.^[40]^ As shown in Figure 3a, upon addition of Zn(II), the colorless spiropyran (SP) transforms into an orange‐colored MC metal complex **1**. Also, a clear absorption band at 504 nm for the complex **1** was evident using ultraviolet (UV) absorption spectroscopy. When the solution was irradiated under visible light for 1 min, the complex **1** switched back to the SP state (Figure 3a). A series of spiropyran compounds were further developed by modifying the various substituents, leading to different metal ion sensors.^[42]^

Most of the further spinopyran metal complexes are designed to stabilize the MC structure by chelation of the metal ion with the ligand while the stability of the SP state is not taken into account. However, balancing the stability of the MC and SP states is crucial for the construction of bistable smart complexes. On the other hand, these spiropyran metal complexes often require high‐energy UV light to drive the photoisomerization process, which leads to poor fatigue resistance. To address these issues, our group has recently synthesized two new spiro‐pyran derivatives (**2** and **3**, Figure 3b) via a novel design strategy.^[41]^ We first grafted an electron‐donating group at the 6‐position of the chromium part to stabilize the SP form, then introduced an ester group at carbon C8 (ortho to oxygen) at the pyran half to form a bidentate ligand to improve the stability of the MC form. Both the spiropyran derivatives are UV‐resistant and were converted to open structures by addition of zinc ions to form red and purple spiropyran complexes (**2‐Zn** and **3‐Zn**, Figure 3c), respectively. The complexes **2‐Zn** and **3‐Zn** exhibit negative photochromism in which the MC‐Zn coordination bond breaks under white light irradiation, resulting in a rapid reversion from the MC to the SP state accompanied by color fading. The fading rate *k* and half‐live of **2‐Zn** were 0.2964 h^−1^ and 2.34 h, respectively (Figure 3d). Its recovery rate after light exposure was 3.3 h. This indicates that both SP and MC have good stability. Also, we showed that moderate‐strength ligand bonds are the key to constructing bistable spiropyran complexes without the need to use UV light by changing the metal ions or ligands.

**FIGURE 4:**
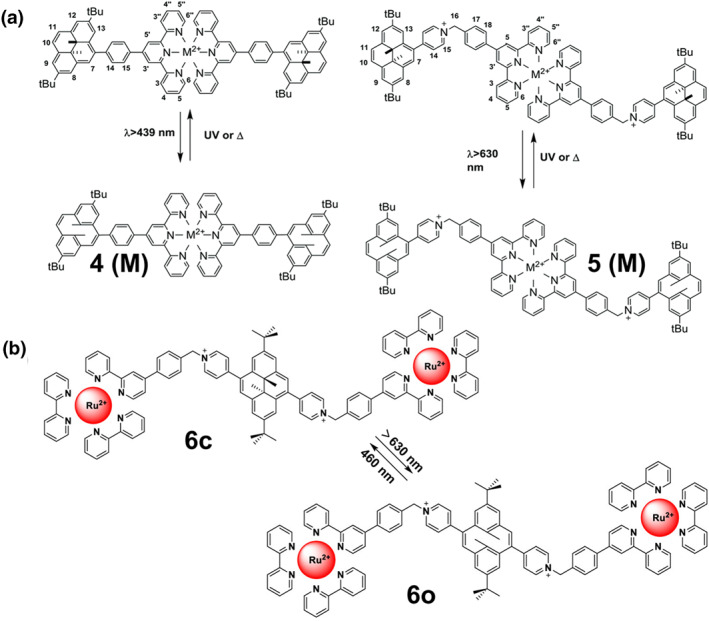
(a) Photoconversion of complexes **4(M)** and **5(M)** (M = Fe^2+^, Co^2+^, and Zn^2+^). Reproduced with permission: copyright 2016, Royal Society of Chemistry.^[43]^ (b) Photoisomerization processes of complexes **6**, Respectively, Reproduced with permission: copyright 2020, American Chemical Society.^[44]^

Similarly, derivatives based on dimethyldihydropyrene also exhibit poor stability and undergo rapid UV degradation, limiting their practical application. This classical negative photochromic compound exists in two isomers, dimethyldihydropyrene (DHP, “closed”) and cyclic arylene diene (CPD, “open”), where the colorless CPD isomer is thermodynamically unstable and transforms to the closed form (DHP) upon heating. Rational bridging of DHP/CPD with metal complexes proved to be an effective solution to these problems. This is because the resulting photoresponsive complexes are able to undergo ring‐opening reactions under visible light irradiation and significantly increase the quantum yield. For instance, Cobo's group has reported a series of metal complexes containing bispyridyl dimethyl dihydro pyrene (**4**(**M**) and **5**(**M**), M = Fe^2+^, Co^2+^, and Zn^2+^), in which the photochromic DHP and the metal complexes are bridged by pyridine or phenyl (Figure 4a).^[43]^ The DHPs undergo ring‐opening reactions under visible light irradiation. The half‐lives of all compounds are about 1.5 h at 318 K and more than 8 h at 298 K. To further enhance the UV resistance properties, a unique all‐visible system was recently introduced by Jacquet et al., which incorporated a DHP photochromic center.^[44]^ In this innovative system, DHP was ingeniously linked to a bipyridine unit or a [Ru(bpy)_3_]^2+^ complex (**6**) (Figure 4b) via pyridine bridging, creating a dynamic and efficient assembly. The DHP undergoes a ring‐opening reaction to form CPD under red (>630 nm) light and a reverse conversion under blue (460 nm) light. For complex **6**, the photostable state of the closed‐loop form increases from 80% to 94%, which is due to the introduction of the [Ru(bpy)_3_]^2+^ in **6**, which acts as a photosensitizer and undergoes MLCT transitions while filling the triplet state of the DHP core.

**FIGURE 5:**
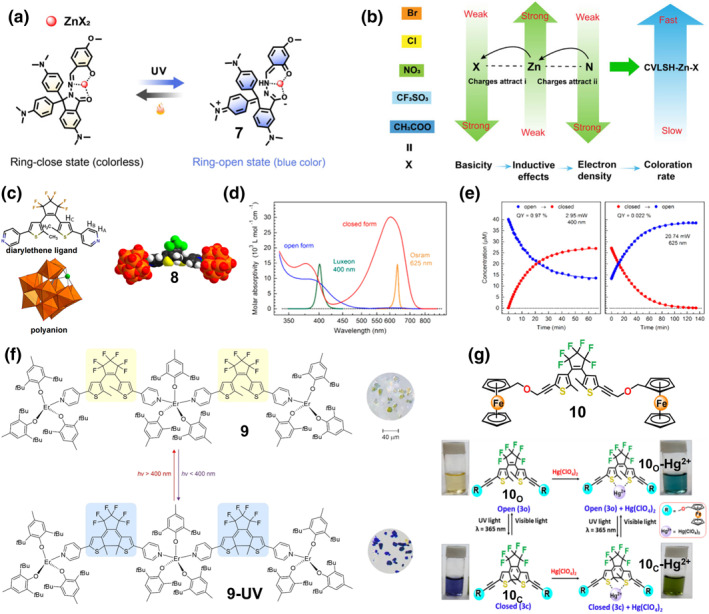
(a) Reversible photo‐induced ring‐opening and ring‐closing reactions for complex **7** and (b) the effect of different counterions on the coloration rates of complex **7**. Reproduced with permission: copyright 2020, AAAS.^[47]^ (c) Chemical structure of the pyridyl‐containing diarylethene ligand, the polyanion and complex **8**, in which the coordinatively unsaturated Co(III) ion of complex **8** is shown as a green sphere. (d) Molar absorptivities for **8o** and **8c** extracted from ^1^HNMR data and the emission profiles, (e) Time‐dependent UV–vis spectroscopy showing the photochemical conversion of **8o** to **8c** and vice versa in response to irradiation with 2.95 mW of 400 nm light and 20.74 mW of 625 nm light. Reproduced with permission: copyright 2018, American Chemical Society.^[48]^ (f) The switching process between complexes **9** and **9‐UV** (left) and their photographs (right). Reproduced with permission: copyright 2022, American Chemical Society.^[49]^ (g) Chemical structure of photochromic molecule **10** and the photoresponsive switching process of **10**_**O**_ and **10**_**C**_ in the presence of Hg(ClO_4_)_2_ or lights of different wavelengths. Reproduced with permission: copyright 2021, American Chemical Society.^[50]^

As mentioned above, the phenolic oxygen atom in the MC form of spiropyran has the advantage of directly coordinating with metal ions, but this form is susceptible to environmental influences. Spironolactone, a novel photoresponsive switch, suffers from the same problem because exposure to negatively charged nitrogen atoms leads to an unstable ring‐opened form.^[45]^ Kopelman et al. proposed an effective strategy for stabilizing ring‐opened isomers by reducing the negative charge density of the nitrogen atom by complexing the metal with the spirooxazine ligand.^[46]^ Further, Zhao's group uncovered a method to modulate the photochromic behavior of spirolactone‐containing complexes by altering the charge density around the spiro‐C‐N moiety.^[47]^ They synthesized spirolactone‐based dyes (L7) and developed zinc complexes with different counter ions (complex **7**:L7‐Zn‐X, X = CH_3_COO^−^, CF_3_SO_3_^−^, NO_3_^−^, Cl^−^, and Br^−^) (Figures 5a,b), where the colorability and coloration rate could be well tuned. The reversible properties of metal coordination bonds between Zn^2+^ and spirolactone‐based ligands were utilized to achieve dynamic modulation of the photochromic behavior.

In comparison with spiropyran derivatives, DAEs and DTEs have unique advantages in areas such as sensors, logic gates, and photoresponsive triggers.^[51]^ Xu et al. report the first photochromic polymetallic oxalate (POM)‐based DAE (DAE) coordination complex **8**.^[48]^ It was prepared by linking two cobalt(III)‐bound borotungstates [B^III^W^VI^_11_O_39_Co^III^]^6‐^ with pyridine‐containing DAE (C_25_H_16_N_2_F_6_S_2_) (Figure 5c). As determined by UV absorption spectroscopy (Figure 5d), both the ligand DAE and polyanion POM do not have strong absorption in the visible band but have strong absorption in the UV band due to the π‐π * (*ε* = 31,500 M^−1^ cm^−1^ at 297 nm) and ligand‐to‐metal charge transfer (LMCT) O_2p_→W_5d_/Co_3d_ electron leaps (*ε* = 32,900 M^−1^ cm^−1^ at 249 nm), respectively. In contrast, complex **8** has a very significant absorption peak in the visible range (400 nm, *ε* = 8702 M^−1^ cm^−1^), which can be attributed to the ^1^A_1_→^1^T_2_ d‐d transition. Such a red‐shifted absorption spectrum of complex **8** with respect to the ligand DAE suggests that the ring closure after molecular assembly can be achieved by a lower energy pathway as compared to uncoordinated ligands. Due to the perturbation of the POM coordination by the DAE electronic structure, the complex **8** can switch between the two states in the visible light. As shown in Figure 5e, the reversible photochemical transitions between **8o** and **8c** are demonstrated by time‐dependent UV‐vis spectroscopy, irradiated with 2.95 mW of 400 nm light and 20.74 mW of 625 nm light, respectively. However, **8o** exhibits a relatively low quantum yield of 0.97%, reaching a photostable state with only 34% **8o** component. This may be due to the fact that the coordination polyanion has a significant electron‐withdrawing capacity that would alter the electron density of the two activated carbons involved in the ring‐closing process and that the stiffness of **8o** may affect the internal rotation required for bond formation. Since the two photoisomers of DAEs can transmit magnetic interactions in different ways and enable control by influencing the coordination geometry of paramagnetic substances, it is widely used to construct photochromic ligands for photomagnetic assemblies. In this context, Rogacz et al. prepared two photoresponsive magnet complexes (**9** and **9‐UV**) using a DAE photochromic ligand bridged with a paramagnetic metal‐centered erbium(III), taking into account both magnetic and photochromic properties.^[49]^ The trinuclear compound **9**, transforms from the open form to its closed form (**9‐UV**) under near‐UV irradiation, accompanied by a color change from yellow to dark blue, which can be reversed under visible light (Figure 5f). The combination of a low coordination lanthanide single‐molecule magnet with a photochromic ligand retains both the desired functionalities (slow magnetic relaxation and photoswitching properties). It should be noted that this photomagnetic effect is irreversible due to the loss of crystallinity of the compounds.

The above design strategy of these DAE derivative‐based photoresponsive complexes mainly involves attaching fluorophore‐containing heteroatoms to the DAE units, and these heteroatom‐containing fluorophores assist or participate in metal coordination, while the metal ions do not attach to the sulfur atoms of the DTE cores themselves, and are not directly involved in the photoisomerization process. In contrast, Karmakar et al. designed a unique DTE‐based photo‐responsive complex **10‐Hg** ^**2+**^ (Figure 5g) in which the Hg^2+^ ions are directly coordinated to the S atoms of the thiophene ring of the photochromic unit, thereby directly interfering with the kinetics of the photoisomerization process.^[50]^ The DTE‐based photo‐responsive molecule **10** exists in two photoisomerization states, **10**_**O**_ and **10**_**C**_, which can be interconverted under UV and visible light irradiation. When Hg^2+^ ions are added, the color of the **10**_**O**_ **‐Hg** ^**2+**^ solution changes from light yellow to blue, while the color of the **10**_**C**_ **‐Hg** ^**2+**^ solution changes from purple to green. Interestingly, interconversion between the two complexes was also possible under UV and visible light, suggesting that the process of metal binding does not interfere with the inherent photoisomerization properties of the DTE core (Figure 5g). However, the photoconversion rate of this Hg^2+^ complex is reduced as compared to the ligand. Kinetic studies by UV‐visible spectroscopy also showed that the conversion rate of free **10o** to **10**_**C**_ (*k* = 0.01 s^−1^) was faster than that of [**10**_**O**_ **‐Hg** ^**2+**^] to [**10**_**C**_ **‐Hg** ^**2+**^] (*k* = 0.007 s^−1^). By theoretical calculations, this can be explained by the fact that the binding equilibrium in ligand **10**_**C**_ is not as stable as that in the open isomer and may form a strained and puckered five‐membered C–S–Hg–S–C ring with Hg^2+^. Quantum yield (Φo→c) results also demonstrate the slower ring‐closing kinetics of **10o‐Hg** ^**2+**^ with a photocyclized quantum yield (Φo→c) of 0.068, which is 8.2‐fold lower than that of the free ligand. This phenomenon suggests that the rate of photoisomerization can be regulated by using appropriate metal coordination to the DTE core to stabilize the ring‐opening state. This design by direct chelation of metal ions within the molecular structure of the DTE core may open new avenues for the development of smart molecular switches for photocontrolled metal coordination.

### 3.2 Photoluminescent bistable coordination complexes

Photoluminescent (PL) complexes with tunable and switchable emissions are prospective smart materials.^[52]^ Lanthanide complexes are outstanding fluorescent materials with high luminescence quantum yields, long excitation lifetimes, large Stokes shifts, and narrow emissions.^[53]^ Therefore, they are widely used as smart fluorescent materials for dynamic anti‐counterfeiting and antioxidants. For example, Eu^2+^/Eu^3+^ is used as luminescent anti‐counterfeiting labels on euro banknotes.^[54]^

**FIGURE 6:**
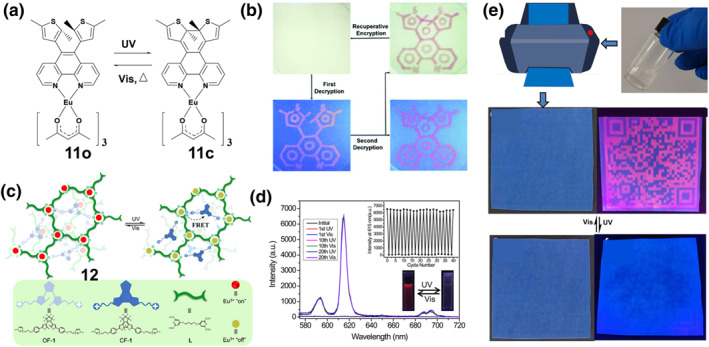
(a) Chemical structure and photochromic reactions of complex **11**, (b) Filter paper under visible light encrypted with Lo and **11**_**O**_ inks. Reproduced with permission: copyright 2016, Royal Society of Chemistry.^[55]^ (c) The chemical structures of complex **12** and its photochromic process; (d) Luminescence emission changes of complex **12** upon consecutive alternating exposure to UV and visible light. Insets show corresponding intensity changes of complex **12** at 615 nm (upper) and their photographs under 254 nm UV lamp (lower). (e) Schematic illustration of the pattern printing process. Light triggered quick response (QR) code with visible/invisible transformation behavior was achieved by using complex **12** as the smart ink. Reproduced with permission: copyright 2021, Springer Nature.^[56]^

However, conventional anti‐counterfeiting materials typically utilize a single‐mode stimulus‐responsive mechanism based on colorimetry or fluorescence, which are limited by spectral overlap and background interference, and have a very narrow range of recognizable identities with low security. In 2016, our group reported a novel europium(III) complex **11** with both photo‐pigmentation and photo‐fluorescence properties, which was obtained by coordinating Eu^3+^ with 5,6‐dithiophene‐1,10‐phenanthroline (L) and acetylacetonate (acac) as ligands (Figure 6a).^[55]^ The open conformation of the prepared **11**_**O**_ is highly emissive owing to the effective energy transfer from ligands to Eu^3+^ ions, but colorless. As the acac co‐ligand is not a sensitizer of Eu^3+^ emission, the resulting ring‐closed conformation **11**_**C**_ is not emissive but shows a red color. Interestingly, this transition can be reversed with irradiation under visible light (*λ* = 530 nm) for 30 min or under natural light for 1 day. Since the absorption spectra of the ligand and complex **11** are identical however, only **11**_**O**_ is emissive, while Lo (the open conformation of L), Lc (the ring‐closed conformation of L) and **11**_**C**_ are non‐emissive. Based on this premise, we used the complex **11**_**O**_ inks and ligand (Lo) inks to draw cryptograms (schematic representation of the L structure) on filter paper. The complex inks were used to draw the structure of Lo, while the ligand inks were used to map the dynamic C‐C bonds to obtain the structure of Lc (a true cryptogram). No information was presented on the filter paper under visible light. After a short period of UV irradiation, the image with the structure of Lo appeared immediately due to the strong red emission (Figure 6b). After prolonged UV irradiation, the true code (an image of the dynamic C‐C bond) appeared as a result of the structural transition from Lo to Lc, while the fluorescence of the **11**_**O**_ ink gradually decreased (Figure 6b).

In order to further construct new smart anti‐counterfeiting materials with higher security and greater difficulty in decryption, combining lanthanide complexes with photoswitches is considered to be an effective method. The fluorescence resonance energy transfer (FRET) mechanism between the photoswitching acceptor and the lanthanide donor is favorable for obtaining special reversible absorption/luminescence modulation. For example, Li and colleagues constructed light‐responsive supramolecular coordination polyelectrolytes (SCPs) **12** through a layered self‐assembly of lanthanide ions, bilayers and diarylethene units.^[56]^ As shown in Figure 6c, the SCP **12** was prepared by mixing Eu^3+^‐L with OF‐1 at a charge stoichiometry (Eu^3+^:OF‐1 = 1:1.5), in which the open‐loop/closed‐loop photoisomerization of the diarylethene controls the FRET process with the lanthanide luminescence center, resulting in reversible luminescence switching. Notably, this diarylethene derivative has bistable properties with an excellent half‐life (*t*_*1/2*_) (estimated at 376.7 min at 298.15 K) (Figure 6c). Meanwhile, **12** exhibited good thermal stability, and no signs of thermal ring opening were observed at high temperatures (333.15 K) in the dark. In particular, the light‐controlled luminescent switch **12** has excellent reversibility and fatigue resistance, and its luminescence intensity did not decrease significantly (less than 4%) even after 20 cycles of alternating UV and visible light irradiation (Figure 6d). Various high‐resolution quick response codes were printed on commercial blue polyester terephthalate (PET) films using the aqueous solution of **12** as the ink. Since the solution is colorless, the PET film has no information under white light. After irradiation with a 254 nm UV lamp, a bright red luminescent pattern appeared and the coded information could be retrieved quickly and accurately by scanning with a smartphone (Figure 6e).

**FIGURE 7:**
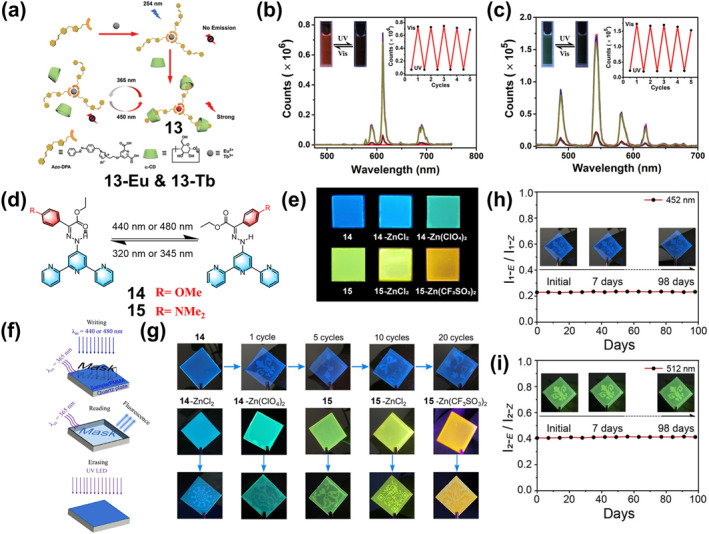
(a) Schematic illustration and molecular structures of complex **13‐Eu** and **13‐Tb**; Photoluminescence spectra and emission intensity of (b) complex **13‐Eu** and (c) **13‐Tb** upon consecutively alternating exposure to UV and visible light. Reproduced with permission: copyright 2022, John Wiley and Sons.^[57]^ (d) Light‐induced E/Z isomerization of hydrazones **14** and **15**. (e) Photos of transparent thin films prepared by drop‐casting with hydrazones **14**, **14**‐ZnCl_2_, **14**‐Zn(ClO_4_)_2_, **15**,**15**‐ZnCl_2_ and **15**‐Zn(CF_3_SO_3_)_2_ under 365 nm UV light; (f) Schematic illustration of writing and erasing patterns on the drop‐casted films using photomask with different light irradiation. (g) Printing complex patterns on the various transparent films with different emission colors. Printed patterns **14** (h) and **15** (i) were kept for more than three months without emission changes. Reproduced with permission: Copyright 2022, John Wiley and Sons.^[58]^

Azobenzene and its derivatives are also photoswitchable with good chemical tunability and photoisomerization efficiency, but they cannot form PL switchable luminescent materials with lanthanide complexes as easily as the examples mentioned above. This is because the excited fluorophore is deactivated by an ultrafast conformational change around the central N=N bond, and therefore most common azobenzenes are non‐fluorescent. In contrast, azobenzene derivatives coordinated with heteroatoms or luminescent lanthanides emit fluorescence but lose their reversible photochromic properties. In order to solve this problem, Yu et al. constructed a photoswitchable luminescent supramolecular system [(Ln^3+^@Azo‐DPA)@α‐CD,**13**] by introducing lanthanide complexes into α‐cyclodextrins (α‐CD) and azobenzene complexes.^[57]^ As shown in Figure 7a, the complex **13‐Eu** solution formed by the azobenzene derivative with the metal ion Eu^3+^ has no fluorescence emission upon excitation owing to the severe dissipation of the excited state energy by the incorporation of the azobenzene unit into the lanthanide complex. After complexing the lanthanide with α‐CD, the strong luminescence of the lanthanide complex was restored due to the non‐radiative pathway of the molecularly rotated *trans*‐azobenzene being suppressed. The photoluminescence of supramolecular lanthanide complexes (**13‐Eu and 13‐Tb**) can be reversibly regulated under light irradiation at different wavelengths due to photoisomeric association and dissociation between α‐CD and azobenzene (Figures 7b,c). The half‐lives (*t*_*1/2*_) of the cis‐azobenzene ligand (Azo‐DPA) and its cyclodextrin host‐guest complex (Azo‐DPA)@α‐CD) at room temperature were 15.1 and 14.9 days, respectively. In contrast, the half‐life of the complex **13‐Eu** was decreased to 6.7 days, which was attributed to the presence of lanthanide ions to promote the *cis*‐*trans* isomerization reaction and accelerate its fluorescence recovery.

In addition to the introduction of lanthanide metal ions, for some luminescent systems with “donor‐acceptor” structure, chemical modification to the ligands and the introduction of transition metal ions to regulate the CT are also effective in realizing PL materials.^[59]^ For example, Ma et al. recently achieved different fluorescence by using the coordination of different zinc salts to the tripyridine moiety to change the electron‐withdrawing ability of the tripyridine portion.^[58]^ They designed and synthesized two novel hydrazone‐based photoswitches **14** and **15** (Figure 7d), in which anisole and N, N‐dimethylaniline (electron donors) were used as rotors and the tripyridyl group (electron acceptor) as a stator. When these two fluorescent switches were combined with different zinc salts, the electron‐withdrawing capacity of the stator portion was altered, resulting in a wide range of emission colors such as blue, cyan, green, yellow, and orange (Figure 7e). The isomeric transition between E and Z for all zinc complexes is accompanied by a switch in fluorescence. Thus, light‐switching molecules with emission colors ranging from sky blue to cyan, green, yellow, and orange can be easily obtained by simply coordinating different zinc salts with **14** and **15**. As shown in Figure 7f, different light irradiations were utilized to achieve pattern writing (15 min of 440 nm irradiation) and erasing (3 min of 365 nm light irradiation) using a mask. The transparent film prepared from zinc‐coordinated hydrazone enables repeated printing of cyan, green, yellow, and orange patterns (as shown in Figure 7g). Importantly, thanks to the excellent bistability of the hydrazone switch, the readability of the printed patterns can be maintained for at least 3 months or more, under ambient conditions (Figures 7h,i).

**FIGURE 8:**
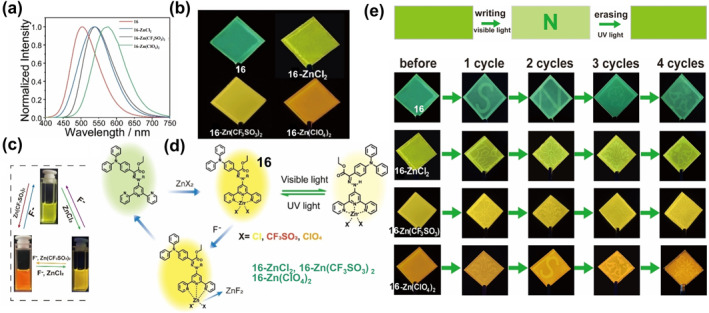
(a) The normalized photoluminescent (PL) spectra of the films of **16**, **16‐ZnCl**_**2**_, **16‐Zn(CF**_**3**_ **SO**_**3**_ **)**_**2**_, **16‐Zn(ClO**_**4**_ **)**_**2**_), and (b) its photos excited at 365 nm. (c) The picture of dynamic fluorescence change in solution. (d) Schematic representation of the dynamic regulation of fluorescence wavelength and intensity based on reversible metal‐ligand (M‐L) coordination and photoisomerization. (e) Schematic illustration of writing and erasing patterns on the films using photomask with alternative visible light and UV light irradiation. Photographs of various films prepared by ligand **16** and these complexes traced under 365 nm excitation. Reproduced with permission: Copyright 2023, John Wiley and Sons.^[60]^

To advance the design and synthesis of precise and selective photochromic compounds for efficient light modulation and signal conversion. Recently, they synthesized a range of complexes incorporating hydrazone compounds with triphenylamine (TPA) and terpyridine push‐pull structures.^[60]^ Initially, they synthesized ligand **16**, followed by the coordination of the ligand with various zinc salts to obtain a series of PL complexes (**16‐ZnCl**_**2**_, **16‐Zn(CF**_**3**_ **SO**_**3**_ **)**_**2**_, and **(16‐Zn(ClO**_**4**_ **)**_**2**_). The films derived from ligand **16** and complexes **16‐ZnCl**_**2**_, **16‐Zn(CF**_**3**_ **SO**_**3**_ **)**_**2**_, and **16‐Zn(ClO**_**4**_ **)**_**2**_) exhibited fluorescence in green, yellow‐green, yellow, and orange at wavelengths of 500 nm, 540 nm, 548 nm, and 574 nm, as depicted in Figures 8a,b. These compounds can repeatedly modulate the emission intensity by undergoing isomerization transitions between the Z‐ and E‐types upon alternating exposure to visible and ultraviolet light, effectively “turning off” and “turning on” the emission. Furthermore, fluoride ions regulate the interactions between metal ions and ligands, enabling dynamic coordination of the ligands with various zinc salts, effectively regulating the emission wavelengths and intensities, as illustrated in Figures 8c,d. Additionally, they fabricated luminescent labels with diverse fluorescent colors utilizing these ligands and complexes, yielding printed images that demonstrated noteworthy stability and flexible dynamic control of fluorescent colors, as shown in Figure 8e.

### 3.3 Light‐driven magnetic switches based on bistable coordination complexes

Magnetic molecules are promising molecular spintronic elements due to their quantized energy levels and the tunability of magnetic properties. Magnetic bistable multifunctional materials have particular potential applications in switching, sensors, and information storage. The widely investigated magnetic bistable systems mainly include spin‐crossover materials, metal‐metal charge‐transfer compounds, valence‐interconversion heterostructure complexes, reversible dimers of organic π‐radicals, and complexes with dynamic coordination environments.^[61]^ These bistable systems have been extensively studied, uncovering new mechanisms for constructing magnetic bistable complexes,^[62]^ such as photochromic molecular magnets based on electron transfer.^[61]^

The metal‐organic complex spin‐crossover (SCO) phenomenon has attracted significant interest in the construction of molecular devices.^[63]^ SCO usually refers to the switching of the spin state between the high‐spin (HS) and low‐spin (LS) ground states of a first‐row transition metal ion with a *d* ^4^ *‐d* ^7^ electronic configuration under external stimuli (temperature, pressure, light and electric fields, etc.), while exhibiting low‐spin (LS)/high‐spin (HS) bistability.[^17^, ^63^, ^64^] A general strategy for constructing photoresponsive magnetic switches consists of inserting photochromic compounds into paramagnetic or ferromagnetic inorganic structures, or designing heterostructures involving photochromic compounds and transition metal complexes. The key to the UV response depends on the structural properties of the photochromic portion, of which the cationic spiropyran salt is a representative compound.[^6^, ^65^] A photochromic cobalt‐bis(dioxene)spirooxazine metal complex, which exhibits both photochromic and redox activity, was synthesized by incorporating photochromic ligands into transition metal coordination complexes by Paquette et al. This strategy provides a new direction for electronic materials for switching and sensor applications.^[66]^

**FIGURE 9:**
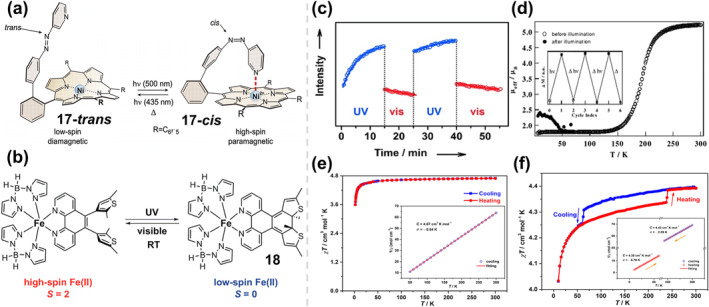
(a) Reversible light‐induced magnetic switching of complex **17**. Reproduced with permission: copyright 2011, AAAS.^[67]^ (b) The chemical structure of complex **18** and reversible photoswitching between paramagnetic high‐spin and diamagnetic low‐spin states. Reproduced with permission: copyright 2013, American Chemical Society.^[68]^ (c) Multiple photoswitching of spin states at room temperature track *in situ* alternating irradiation with UV (*λ* = 282 nm, blue circles) and visible light (*λ* > 400 nm, red circles). Reproduced with permission: copyright 2015, John Wiley and Sons.^[69]^ (d) The *μ*_eff_ versus *T* plots before and after illumination. The sweep rate is 2 K min^−1^. Insert: changes in the magnetization at 5 K. Reproduced with permission: copyright 2002, Elsevier.^[70]^ Temperature dependence of χ*T* for **21** (e) and **22** (f) in the cooling (blue) and heating (red) modes at a direct‐current field of 1000 Oe. Insert: plots of 1/χ versus T for **21** and **22** with Curie‐Weiss fitting. C and *θ* represent Curie temperature and Weiss constant. Reproduced with permission: copyright 2022, Springer Nature.^[61]^

Conventional bistable magnetic materials, however, are often confined to solid materials or very low temperatures. To break through this bottleneck, Herges and co‐workers were the first to achieve bistable molecular spin switches in homogeneous solutions at room temperature in 2011.^[67]^ They prepared a reversible photomagnetic switch based on an azopyridine‐functionalized nickel porphyrin, in which the coordination number of the nickel(II) porphyrin complex **17** was altered by *cis*‐*trans* isomerization of the azo compound, thereby changing the spin state (Figure 9a). The formation of coordination bond between the pyridine ligand and the nickel center was induced by irradiation with blue‐green light (500 nm) accompanied by the electronic rearrangement from the antimagnetic to the paramagnetic state. This process can be reversed upon irradiation of violet‐blue light (435 nm). In the *cis*‐isomer, the pyridine unit is coordinated to the nickel center, which has a coordination number of 5 and an open‐shell electronic structure (*S* = 1). However, in *trans‐*isomer, the pyridine unit is too far away to participate in the coordination, and the nickel center is only coordinated to the porphyrin, which has a coordination number of 4 and a closed‐shell electronic structure (*S* = 0). Of particular note is that it is bistable in homogeneous solutions at room temperature, with the *cis*‐azopyridine isomerizing into a more stable trans conformation with a half‐life of several hours, while the **17‐*cis*** is stable for several weeks (25% isomerization in 10 weeks at 294.15 K; half‐life of 27.2 h at 327.15 K). **17‐*cis*** is even more thermally stable in DMSO (5% isomerization in 3 days at 343.15 K). No degradation or fatigue was detected after more than 10,000 cycles, indicating that the photo‐optical light has good fatigue resistance.

Similarly, the integration of diaryl vinyl ligands into spin‐crossover molecules is an efficient way to achieve magnetic spin‐crossover metal complexes. Khusniyarov and colleagues obtained photo‐triggered magnetic switches **18** by incorporating divinyl ligands into SCO iron (II) complexes containing boron‐bridged pyrazolyl ligands (Figure 9b).^[68]^ The photoactive ligands were utilized to create two configurations with different ligand field strengths under different wavelength irradiation, resulting in different spin preferences, that is, *S* = 2 for the open form and *S* = 0 for the closed form. Both forms are thermally stable at room temperature with a semi‐solubility time of 18 days in solution.

In order to be integrated into devices, molecular switches must function optimally within specific environmental parameters, namely at room temperature and atmospheric pressure. In contrast to systems exhibiting thermally induced memory effects, the optical resolution of spin states proves more appealing owing to its swift response time, minimal power consumption, and heightened selectivity. Current approaches include the creation of bistable CPs capable of undergoing photo‐induced phase transitions at room temperature, as well as the utilization of the light‐induced excited spin‐state trapping effect. Nevertheless, the former lacks the capability for single‐molecule‐level switching, thereby hindering the utilization of established bistable bulk‐phase materials and nanomaterials in contemporary techniques for SCO switching. The latter, on the other hand, although achieving true single‐molecule‐level optical switching, usually requires temperatures below 50 K due to the rapid relaxation of the photoinduced high‐spin state to the low‐spin ground state in nanoseconds at room temperature. Consequently, controlling the magnetic properties at the molecular level remains a pivotal challenge in the development of functional SCO devices. Viable approaches to address these challenges include incorporating photoisomerizing ligands into bistable molecules, enabling remote initiation of metal center SCO. This method has been demonstrated in ligand‐driven photoinduced spin changes and light‐driven ligand‐induced spin state transitions. Also, previous reports of photoswitching via ligand‐driven effects have predominantly concentrated on the liquid phase or reported strictly one‐way photoswitching in the solid state, which is still a long way from practical applications. Hence, in 2015, their group proposed a photo‐reversible solid‐state spin‐crossover molecular complex at room temperature, that is, iron (II) complexes with photoisomeric DAE ligands.^[69]^ After exposure to UV light (*λ* = 282 nm) for 12 h at room temperature, this complex switches from paramagnetic HS state to diamagnetic low‐spin state (HS→LS, 68% HS and 32% LS) due to the formation of a stable closed‐loop isomer of the DAE ligand. The LS to HS transition (84% HS and 16% LS) occurs after 8 h of visible light irradiation (*λ* = 400 nm). They further carried out photoswitching experiments by recording near‐edge X‐ray absorption fine structure spectra under alternating UV and visible in situ irradiation, as the intensity of the Fe 2p_3/2_→e_g_ transition (710.2 eV) can serve as a probe of the LS content. As shown in Figure 9c, the LS content increased significantly after UV irradiation, while the LS content decreased after visible light irradiation, suggesting that the complex has a good light‐induced HS→LS→HS transition property. This is the first clear example of molecular‐level reversible SCO in the solid state at room temperature.

Within hybrid structures comprising both molecular and inorganic materials, a critical challenge lies in preserving specific functionality when utilizing fragile building blocks like molecules to fabricate novel molecular devices. Despite notable advancements in recent years in preparing thin/thick films and nanogaps using magnetic spin‐crossover metal complexes, the switchability of these materials has remained constrained to extremely low temperatures or cooling–warming cycles. According to the aforementioned example, photoinduced SCO conversion has been observed at room temperature through the ligand‐driven light‐induced spin change (LD‐LISC) effect.[^63^, ^69^] The photoinduced states in the ligand‐driven effects exhibit stability comparable to the photoisomers of the ligand, with lifetimes of up to several years at room temperature. The molecular spin‐crossover Fe(II) complex, featuring photochromic diaryl vinyl ligands, namely [Fe^II^(H_2_B(pz)_2_)_2_phen*] (complexes **19**, pz = 1H‐pyrazol‐1‐yl, phen* = a diarylethene‐functionalized phenanthroline ligand), has been successfully demonstrated to undergo reversible switching between HS and LS states at room temperature in both solution and the solid state.[^68^, ^69^] Molecular‐level photoswitching is initiated through the reversible photocyclization of the diarylethene‐based ligand, resulting in a photoconversion from HS to LS states at room temperature. Consequently, the prospect of transferring this distinctive iron(II) molecular photoswitch onto surfaces is highly tempting. Based on this, Poggini et al. dedicated their efforts to obtain 5 nm films that maintain photomagnetic switchability at room temperature and the creation of benchmark tools for future nanoscale SCO studies.^[71]^ They prepared nanoscale ultrathin films (5 nm) corresponding to about 6–8 monolayers of complexes **19**, by a thermal evaporation process under ultrahigh‐vacuum sublimation on Au(111) substrates prepared by standard sputtering and annealing cycles. Photoswitching of nanoscale films with bistable SCO at room temperature was achieved for the first time through remote triggering of the SCO of ferric ions by photocyclization of divinyl ligands. The authors employed X‐ray photoelectron Spectroscopy (XPS) in the Fe2p region to monitor the SCO phenomenon in the films. The confirmation of thermally induced SCO in the films was supported by evident linear changes in the Fe2p spectra, the precise positioning of extracted components, and their corresponding intensities. In contrast to the pristine powder samples, the nanostructures have a strong effect on the compounds, in particular inducing a slower SCO transition and increasing the residual HS fraction (incomplete SCO) at low temperatures. These observations can be tentatively attributed to structural inhomogeneities and a limited degree of elastic interactions among interconverted molecules in the film, resembling the conditions observed in previously studied doped SCO systems. They further investigated the switching of the same 5 nm films by in situ XPS with UV irradiation at RT. Exposure to UV light (*λ* = 282 nm) for 8 h measured at 300 K revealed that the evolution of the Fe2p region is very similar to that observed during the thermally induced HS‐to‐LS transition. The increase in the LS fraction implies that a stronger ligand field at the ligand Fe(II) ions, instigating the HS‐to‐LS transition (LD‐LISC effect). The LS contribution nearly doubled to 8.9% after UV irradiation, in contrast to the original film measurement at RT, which was 5.1%. This is because UV‐induced cyclization of the open‐ring isomer (phen*‐o) results in the formation of the closed‐ring isomer (phen*‐c). The films exposed to UV radiation exhibited an additional component (∼25 ± 5%) at a lower binding energy (164 eV), corresponding to the binding energy associated with phen*‐c. This suggests that the photoinduced SCO is activated at a distance through the photoreaction of the phen* ligand. As reported previously, the spin‐spin transition remains “locked” in the high‐spin state due to the existence of cooperative effects or intermolecular interactions in bulk materials.^[63c]^ Thin films prepared by thermal deposition, on the other hand, can exhibit both thermal and photo‐induced spin transition behaviors without the influence of solid‐state effects, such as cooperative or intermolecular interactions. However, the molar fraction of photo‐induced conversion of Fe(II) in nanoscale ultrathin film is lower than the fraction of photoswitching in solution and solid. Although ultrafilms are believed to alleviate the penetration depth issue, the lower efficiency observed in these films may suggest either a diminished fraction of photoreactive antiparallel conformations at the nanoscale or undesirable filling effects within the films.

In addition to the SCO phenomenon, many redox‐active complexes can exhibit valence‐interconversion isomeric behavior due to the interconversion between two valence isomers induced by heat or light due to intramolecular CT.^[72]^ As a classical example, Fujishima's groups describe a novel Co compound **20‐Co** ^**III‐LS**^ (the compound 20‐Co^III‐LS^ is an abbreviation for [Co^III‐LS^(tmeda)(3,5‐DBSQ)(3,5‐DBCat)]·0.5C_6_H_5_CH_3_, where LS, tmeda, 3,5‐DBSQ and 3,5‐DBCat denote low‐spin, N,N,N,N′‐tetramethylethylenediamine, 3,5‐di‐tert‐butyl‐1,2‐semiquinonate and 3,5‐di‐tert‐butyl‐1,2‐catecholate, respectively) that exhibits intramolecular electron transfer with an extremely long lifetime in the visible light response.^[70]^ Due to the LMCT band around the visible region of the covalent reciprocal isomeric compound, a laser with a wavelength of 532 nm was used as a light source. The magnetization values of the complexes increased after light irradiation. The magnetization intensity at 5 K was about 1.7 μB before irradiation and about 2.3 μB after irradiation (Figure 9d). This suggests that an electron in 3,5‐ DBcat is transferred to Co^III‐LS^ under light, producing Co^II‐HS^ and 3,5‐DBSQ. The process is denoted as **20‐Co** ^**III‐LS**^→**20‐Co** ^**II‐HS**^, involving the reduction of quinone to catechol. By measuring the relaxation curves of the magnetization values at 5 and 15 K, it can be found that the rate of decay of the substable state is relatively slow after the light is turned off, with a lifetime of 175 and 70 min at 5 and 15 K, respectively. The light‐induced changes in magnetization intensity can be maintained at low temperatures, and the substable state returns to its initial state after heat treatment above 50 K. These results suggest that the light‐induced change in magnetization intensity can be maintained at low temperatures and has a good reversibility (Figure 9d, inset). Such a photoinduced valence tautomerism is involved in photoinduced magnetization and photoinduced spin transfer.

The width of the thermal hysteresis is crucial in bistable materials. Current strategies for realizing large thermal hysteresis focus on enhancing synergistic interactions between magnetic molecules and extending dimensionality through π‐π superposition and hydrogen‐bonding interactions. Besides spin crossovers and intermetallic CT compounds, valence‐interchange isomeric complexes involving free radicals or reversible dimerization of organic π‐radicals are proposed as effective means to attain magnetic bistability with wide thermal hysteresis loops. While incorporating radicals into molecular magnets proves effective, directly synthesizing stabilized radicals is time‐intensive and involves hazardous redox reactions induced using chemical and electrochemical processes. On the other hand, photochromic materials attract significant attention for generating stabilized radical analogs through electron transfer under ultraviolet‐visible (UV‐Vis) light exposure. For instance, ligands based on anthracene display radical‐driven photochromic behavior, and magnetic interactions transpire between radicals and paramagnetic metal ions. Nevertheless, achieving magnetic hysteresis effects in electron‐transfer photochromic materials remains a formidable challenge. Hu et al. recently introduced a novel photoactive ligand, 9,10‐anthracene dicarboxylic acid (H_2_ADC), aiming to enhance the thermal hysteresis in magnetic bistable systems.^[61]^ They employed a strategy involving the generation of stabilized two‐radical radical‐driven photochromism under xenon (Xe) lamp irradiation. Specifically, a series of isostructured single‐chain complexes [M(ADC)(H_2_O)_2_(DMF)_2_]_n_ (M = Mn^2+^, Zn^2+^, Ni^2+^, and Co^2+^ for **21**, **22**, **23**, and **24**, respectively) were prepared by combining H_2_ADC with transition metal ions. Among them, **21** and **22** show visible photochromic phenomena after xenon (Xe) lamp irradiation. Due to the variation in magnetic coupling between Mn^2+^ ions and photogenerated radicals, compound **21** exhibits a significant photomagnetic response upon light irradiation, resulting in an unprecedented wide thermal hysteresis with a temperature width of up to 177 K (*T*_1/2*↓*_ = 62.7 K; *T*_1/2*↑*_ = 239.8 K; *T*_1/2*↓*_ and *T*_1/2*↑*_ denote the transition during cooling and heating, respectively, during DC magnetization rate measurements temperature) (Figures 9e,f). Single‐crystal analysis reveals that the length and angle of the Mn‐O bond remain constant during cooling, signifying the absence of a spin transition at the metal center. Nevertheless, the ADC ligand, adopting a monodentate coordination mode, undergoes rotation along the Mn‐O2 bond with decreasing temperature, resulting in a dihedral angle of 2.724(2)° between the rings at different temperatures. This rotation induces a modification in the magnetic coupling between the Mn^2+^ ion and the photogenerated radicals, yielding an exceptionally broad thermal hysteresis of 177 K. Radical‐activated photochromic materials achieve an unprecedented magnetic bistability with a large thermal hysteresis backline. The utilization of this photochromic material in crafting comprehensive thermally hysteretic molecular magnet systems will contribute to the convergence of molecular magnetism, electronics, and photonics.

### 3.4 Light‐induced reversible single crystal to single crystal transformation in bistable coordination complexes

The transition from single crystal to single crystal (SCSC) represents a unique type of solid‐state reaction characterized by responsiveness to multiple stimuli (such as solvent, temperature, and light).^[73]^ For instance, exposure of single crystals to light can induce a SCSC transition. The transformation of coordination compounds via single‐crystal to single‐crystal (SCSC) processes may exhibit reversibility or irreversibility. Primary transformation mechanisms encompass: (1) loss or gain of ligand and/or lattice solvent molecules, while retaining or altering the original structure^[74]^; (2) complexation of metal ions^[75]^; (3) reactions involving ligand exchange^[76]^; (4) chemisorption/desorption of gases (more broadly, absorption or release of guest molecules)^[77]^; (5) redox reactions^[78]^; (6) chain isomerization^[79]^; (7) photochemical [2 + 2] cycloaddition reactions of C=C‐bonded ligand‐containing molecules yielding cyclobutane derivatives.^[80]^ These processes frequently entail induced structural modifications, such as alterations in the cores of separate polymetallic complexes,^[81]^ transformation of discrete complexes into CPs,^[82]^ or adjustments in the dimensionality of CPs.^[83]^ Furthermore, alterations in the coordination environments of metal ions and reorganization of crystal packing could give rise to substantial spectral variations (e.g., color, optical activity, or luminescence),^[84]^ and notable modifications in magnetic properties in the presence of paramagnetic centers.^[85]^

Since 1971, when Schmidt first reported the phenomenon of the light‐induced SCSC transition,^[86]^ UV irradiation‐based induced SCSC reactions have received great attention.^[87]^ In most cases, the photoinduced SCSC transition is achieved by [2 + 2] cycloaddition, in which the two reaction centers are closely aligned in parallel (less than 4.2 Å apart).^[88]^ However, [2 + 2] cycloaddition reactions between olefins tend to inevitably undergo photoinerting or molecular sliding.[^83^, ^89^] On the other hand, stilbene may undergo a cyclization photoreaction to generate dihydrophenanthrene and tends to irreversibly convert to phenanthrene by a dehydrogenation reaction upon further exposure to air.^[90]^ The above unfavorable factors tend to result in an irreversible light‐induced SCSC transformation, which is not conducive to the design of smart switches with photoresponsive properties.

**FIGURE 10:**
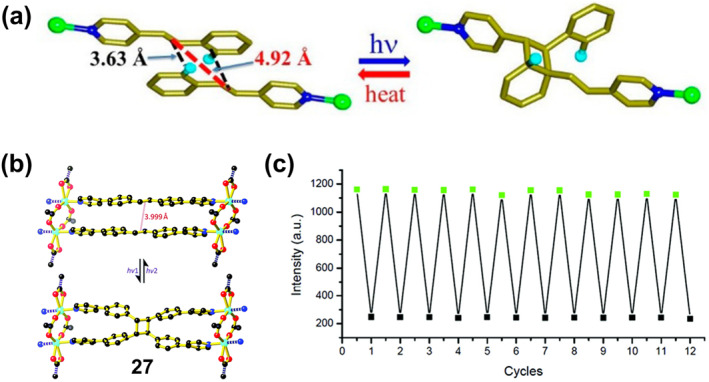
(a) Solid State [2 + 2] photo‐cycloaddition reaction between phenyl–olefin bonds and its reversible cleavage reaction by heating. Reproduced with permission: copyright 2015, American Chemical Society.^[91]^ (b) View of the photoinduced single crystal to single crystal (SCSC) transformation between 4,4′‐dpsb and tppcb in **27** and **27a**, respectively. (c) The cyclization‐cycloreversion reversibility of **27**. Reproduced with permission: copyright 2020, Royal Society of Chemistry.^[92]^

To solve this problem, Medishetty et al. reported a zinc complex that undergoes SCSC transformation under UV light irradiation, namely **25** [Zn_2_(ptol)_4_(4spy)_2_] ((ptol = para‐toluate), 4spy = 4‐styrylpyridine) and **26** [Zn_2_(ptol)_4_(2F‐4spy)_2_] (2F‐4spy = 2‐fluoro‐4′‐styrylpyridine).^[91]^ Since the adjacent 4spy ligands in the crystal are organized in a head‐to‐tail fashion, the normal cycloaddition reaction of the olefin bond of the 4spy ligand occurs on one side of the crystal structure of the complex, while a rare [2 + 2] cycloaddition reaction between the phenyl olefin double bond on the other side occurs, forming a one‐dimensional CP (Figure 10a). Sliding of the 4spy group is limited by the steric hindrance of the adjacent methyl group, and the complex is shown to be reversible under heat treatment.

Alternatively, Li et al. addressed the above problem by constructing interlocking structures of CPs by reducing the distance between neighboring functional groups and stabilizing in situ‐generated radicals.^[92]^ This strategy provides a valuable opportunity for single‐crystal‐to‐single‐crystal (SCSC) conversion. They prepared a bipyridyl‐substituted stilbene, 4,4′‐bis(4‐pyridyl)‐stilbene (4,4′‐dpsb), and constructed interlocked structural CPs using this polydentate pyridine compound. In each pair of nearest 4,4′‐dpsb molecules, they are associated with each other by reversing the center of symmetry, where the distance between the parallel olefin bonds is only 3.999 Å. Such an interlocking structure helps to reduce the distance between adjacent functional groups and stabilize the in situ generated radicals, while the formation of robust CPs facilitates the single‐crystal to single‐crystal (SCSC) transition. After irradiation with UV light (*λ* = 365 nm) for 30 min, crystal **27** {[Zn(1,4‐pda)(4,4′‐dpsb)]n (1,4‐H2pda = 1,4‐phenylenediacetic acid)} transformed to [Zn(1,4‐pda)(tppcb)0.5]n (**27a**) (tppcb = 1,2,3,4‐tetra(4‐(pyridin‐4‐yl)phenyl)cyclobutane, while the transparency and single‐crystal nature of the crystal were preserved, indicating that a SCSC transition occurred (Figure 10b). Following irradiation with short‐wave UV light (*λ* = 254 nm) for 30 min, in which the cyclobutane cleaved into olefin bonds, crystal **27a** underwent a reversible SCSC transition to **27**. Moreover, this coordination network has good fatigue resistance, and the emission intensity of the sample did not decay significantly after 12 cycles of alternating 365 nm light and 254 nm light irradiation for 30 min (Figure 10c). More importantly, states **27** and **27a** in this coordination network exhibit typical fluorescence switching bistability, which is potentially valuable for applications in the field of optical memory.

## 4 THERMALLY RESPONSIVE SYSTEMS

Temperature is one of the most widely used stimuli occupying an important place in the design of stimulus‐responsive smart materials due to its availability, ease of control, and diverse application scenarios (with practical advantages both in vitro and in vivo).^[21d]^ A large range of highly tunable thermal stimulus‐responsive coordination materials can be realized by carefully designing the strength of the coordination bonds. Under thermal stimulation, the ligand bonds or specific covalent bonds break or recombine, inducing structural rearrangements or interconversions and thermally excited proton transfers. Thus, thermally stimulated responsive complexes have found a wide range of applications in the fields of luminescence modulation, color switching, and volumetric phase transitions.^[93]^ In this section, several typical thermo‐responsive materials are summarized, including thermochromic, thermoluminescent, and thermoresponsive magnetic molecules.

### 4.1 Thermochromic bistable coordination complexes

Thermochromic materials based on metal complexes are drawing increasing attention due to their great value in applications such as safety signs and temperature sensors.^[94]^ The thermochromic mechanism primarily encompasses intramolecular electron transfer,^[95]^ functional group conversion of ligands, co‐ligand transformation,^[96]^ as well as alterations in ligand geometry^[97]^ or ligand number^[98]^ A large number of thermochromic systems have been reported, although most are only applicable in the solution state. In order to obtain solid‐state thermochromic systems with greater research and application value, a classical example is the nitro‐nitrito heterostructure in Ni(II)L_2_(NO_2_)_2_ complexes reported by Fabbrizzi and colleagues, where L stands for N‐alkyl substituted ethylenediamine.^[99]^ This Ni(II) bis(diamine) complex has been reported in the literature to exhibit chain isomerization in the solid state under temperature‐driven conditions.

**FIGURE 11:**
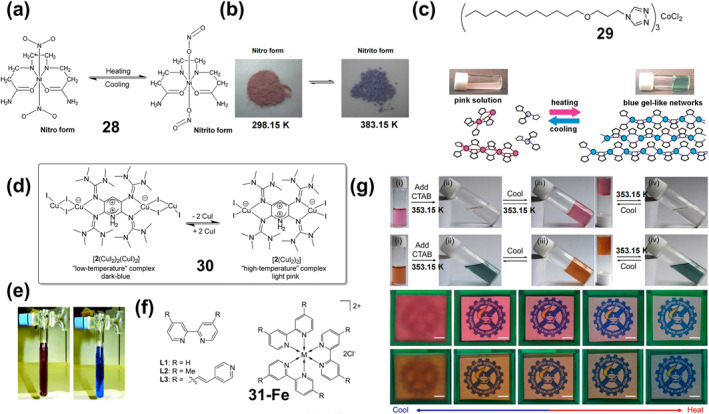
(a) The thermochromic properties of complex **28** and (b) its color change. Reproduced with permission: copyright 2012, Elsevier.^[96]^ (c) chemical structures of lipophilic complex **29** and pictures of complex **29** in chloroform: (left) a pale pink solution at 273.15 K; (right) a blue gel‐like phase at 298.15 K. Reproduced with permission: copyright 2004, American Chemical Society.^[100]^ (d) Reversible thermochromic reaction of complex **30** and (e) their photos in equilibrium at RT and at 233.15 K. Reproduced with permission: copyright 2015, John Wiley and Sons.^[101]^ (f) Chemical structure of the ligands L1–L3 and the complexes **31‐Fe**. (g) Photographs of the thermochromic metal‐ligand (M‐L) system for optical color switching in their gel state. The photos of 10 × 10 cm color switchable window prototypes containing FeCl_2_ or CoCl_2_. Reproduced with permission: copyright 2020, John Wiley and Sons.^[102]^

On this basis, in order to further improve its reversibility, Chao et al. proposed a strategy to promote the reversible isomerization of nitro‐nitrito using co‐ligands (a weak‐linked amide group).^[96]^ They prepared solid nickel (II) diaminodiamine complexes [Ni(C_8_H_18_N_4_O_2_) (NO_2_)_2_]‐H_2_O (**28**) with thermochromic properties, in which the NO_2_^−^ ion is bound to the metal center via a nitrogen atom (Figure 11a). When the filter paper coated with complex **28** was placed close to or away from the heating plate, the color of compound **28** switched rapidly and reversibly between red‐violet and blue (Figure 11b). This is because the vibration amplitude of the chelate ring formed by the diaminodiamide backbone increases when heated, resulting in a decrease in the bonding strength of Ni‐O=C. The weak chain amide group in this system, fortunately, has the effect of maintaining the stability of the compound, which leads to a reduction in the available space for the axially bound anion and promotes the rearrangement of the nitro to the less spatially demanding nitrito coordination mode.

As described in the aforementioned examples, low‐dimensional structural coordination compounds are typically limited to serving as structural motifs for bulk crystalline materials. Meanwhile, achieving thermochromic solution systems with controllable structure and stability is also a challenge. To address this issue, Kimizuka and colleagues developed reversible thermochromic gels characterized by controllable structure and stability through the integration of lipid‐soluble triazole ligands and one‐dimensional complexes.^[100]^ They synthesized lipophilic cobalt(II) complex **29** of 4‐alkylated 1,2,4‐triazoles to form lipid‐encapsulated nanowires(Figure 11c), dispersing halogen‐bridged mixed‐valent platinum complexes in an organic medium. As depicted in Figure 11c, upon dissolution of complex **29** in spectroscopic grade chloroform, a blue gel‐like emerged under ambient conditions. Such a gel‐like structure exhibit long‐term stability over a period of 1 month. Unlike traditional gels, the blue gel undergoes a color transformation to a pale pink solution upon cooling to 273.15 K, and reverts to its original gel state upon re‐heating to room temperature, demonstrating a fully reversible thermochromic transition (Figure 11c). The alteration in color within this gel is attributed to electron transfer between the metal ions and the organic ligands constituting its composition. Upon heating, the metal ions undergo a transition from a lower to a higher oxidation state, leading to the transference of electrons from the organic ligands to the metal ions, thereby altering their absorption spectra and effectuating a color migration. Conversely, upon cooling, the metal ion reverts back to its initial low oxidation state, prompting the redistribution of electrons, and consequently inducing a change in color. Such lipophilic modification of one‐dimensional coordination systems imparts distinctive solution properties that hold potential for broad applications in the development of thermoresponsive self‐assembled molecular wires.

In addition to the intramolecular electron transfer mechanism of thermochromism, a reversibly activated thermochromic Cu^I^ tetrakisguanidine complex **30** based on MLCT bands has been reported by Himmel and co‐workers.^[101]^ The complex is formed by a redox‐active dicationic guanidino‐functionalized aromatic ligand bridging two Cu_2_X_3_^−^ units (X = Br or I). The tetranuclear low‐temperature complex is blue, and after the temperature is increased, the strong MLCT band disappears due to the elimination of CuX groups, resulting in a thermochromic effect leading to a red‐pink binuclear Cu^I^ high‐temperature complex (Figure 11d). After cooling in an isopropanol/dry ice bath, the complex undergoes a significant reversible color change (Figure 11e).

In contrast to the host‐guest interactions related to many natural processes, temperature is not typically considered a precise stimulus for artificial chemical systems, and it is difficult to achieve an adequate recording of bistable thermochromic systems. In response to this challenge, Nirmala et al. ingeniously devised metal‐bipyridine complexes, employing temperature as a precise modulator to realize multi‐state volatile memories and arithmetic operations.^[102]^ This modulation is achieved through a reversible ligand‐exchange reaction between the coordinating bipyridine and excess DMSO. The initial step involved the synthesis of diverse metal complexes, chelating ligands L1‐L3 with first‐row transition metal chlorides (Fe^2+^, Co^2+^, Ni2^+^), dissolved in DMSO (Figure 11f). Different ligands were employed to finely adjust the electron density on the bipyridine nitrogen. These complexes displayed greater stability, reversibility and reproducible optical states, enhanced sensitivity to temperature changes, and a wider operating window (298.15–363.15 K) (**31‐Fe**, **31‐Co**, **31‐Ni**) (Figure 11f). The authors systematically investigated how the ratio of substituent, solvent, ligand, and M‐L influences the thermochromic behavior of various metal ions. Notably, the ligand L2, featuring an electron‐donating methyl substituent, demands a comparatively elevated temperature to bleach the MLCT absorption band within the complex. High boiling point polar solvents facilitate thermochromism, and the use of diverse ligands enables the complexes to exhibit color‐to‐colorless and color‐to‐color transitions upon heating in the ligand solvent. Ultimately, the authors demonstrate that a 1:1 M‐L ratio exhibits reversible thermochromism. Additionally, they finely tuned the M‐L ratios to extend the switching behavior and achieve observable color‐to‐color transitions. Finally, they demonstrated temperature‐dependent color switching in the gel state by mixing cetyltrimethylammonium bromide gels and metal complexes, as shown in the Figure 11g. No degradation of thermochromic properties was observed after several heating‐cooling cycles. Moreover, a smart window with a sandwich structure further demonstrated that this temperature‐responsive color‐changing gel can be easily and reversibly switched between transparent and clear (Figure 11g), displaying good stability without any observable degradation in performance after 1 month. This work provides one of the first demonstrations of the use of temperature as a precise input to affect stable, reversible and reconfigurable optical states in a responsive chemical system with the properties of volatile polymorphic memory and arithmetic data accumulation.

### 4.2 Thermochromic luminescent bistable coordination complexes

Thermochromic light‐emitting materials (TLMs) are a class of materials whose emission wavelengths change reversibly with temperature.^[93b]^ Thermochromic luminescent metal complexes have great promise for applications such as thermometers, bioimaging, displays, optical information recording, and security applications.[^21^, ^59^, ^103^] Currently, there are three main strategies to construct TLMs.^[104]^ First, both organic ligands and metal centers can be used as luminescent regions, and a diversity of luminescence changes can be generated through M‐L CT.[^103^, ^104^] Second, using multinuclear metal clusters containing d^6^, d^8^ and d^10^ electronic structures or ligand polymers with pro‐metal interactions to achieve significant changes in photophysical properties.[^103^, ^105^] Finally, the 4f internal electron conversion of lanthanide(III) ions or the reverse energy transfer of ion‐ligands can be utilized to achieve unique intrinsic optical properties.[^104^, ^106^]

**FIGURE 12:**
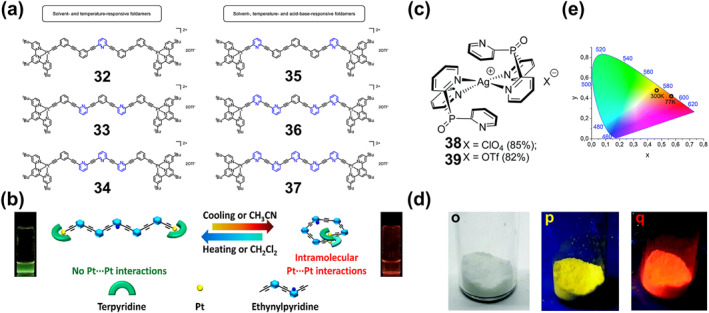
(a) Molecular structures of complexes **32**–**37**, (b) the schematic diagram of the helix–coil transition mediated by solvents or temperatures; Reproduced with permission: copyright 2019, American Chemical Society.^[107]^ (c) The structures of complexes **38** and **39**, (d) The powder of **38** under day‐light at 300 K (o), under UV‐light at 300 K (p) and about 80 K (q); (e) CIE‐1931 chromaticity diagram showing temperature dependence of the emission color of **38** (λex = 350 nm). Reproduced with permission: copyright 2019, Royal Society of Chemistry.^[108]^

Based on these design strategies, more materials with thermochromic fluorescence have been developed, but challenges remain that need to be solved to realize the practical application of these smart bistable complex materials. For example, helical folded body skeletons are important in biologically relevant fields but suffer from poor stability, while in contrast, metal coordination provides helical stability but suffers from limited dynamic folding behavior. Thermoresponsive luminescent platinum complexes have suitable coordination capabilities but their thermoluminescence mechanism is mostly induced by intermolecular interactions. To address this issue, Chan et al. reported a series of single‐component thermally responsive Pt(II) complexes (**32**–**37**) which utilized intramolecular Pt‐Pt interactions (Figure 12a) to impart reversible folding and unfolding properties, resulting in reversible switching in the emission in response to temperature changes (Figure 12b).^[107]^ When the complexes (**32**–**37**) were heated in CH_3_CN, the unfolding process resulting in a shortened distance between the Pt centers and a blue shift in the low‐energy emission band at 646–680 nm due to the weakening of the Pt‐Pt interaction, accompanied by a change in emission color from red to green.

Cu(I) and Ag(I) complexes are promising materials for the preparation of smart systems due to their excellent photophysical properties and structural diversity, while their luminescent properties are responsive to temperature, mechanical forces and volatile organic compounds (VOCs).^[109]^ However, in the vast majority of Cu(I) and Ag(I) complexes, the d^10^ metal cation has a pseudotetrahedral geometry., which makes them prone to geometrical deformation into planar structures and attack by external nucleophilic reagents. This leads to a strong increase in the non‐radiative relaxation rate until the emission quenches completely. Therefore, in order to suppress this emission quenching, the design of complexes with certain structural stiffness ligands is necessary. In this context. Artem'ev et al. reported for the first‐time Ag(I) complexes with square planar geometries (**38 and 39**, Figure 12c), which exhibited significant solid‐state photoluminescence and reversible thermochromic behavior at room temperature via the reaction of tris(2‐pyridyl)phosphine oxide (Py3PO) and Ag(I) salts.^[108]^ As shown in Figure 12d, the emission color of **38** changed from yellow (*λ*_max_ = 585 nm) to red‐orange (*λ*_max_ = 620 nm) when the temperature was reduced from room temperature to 77 K. This thermochromic phenomenon is fully reversible, as shown in Figure 12e, where the heating of sample **38**–300 K leads to a recovery of the emission. Complex **39** shows a similar pattern, reversibly changing the emission color from yellow at 300 K (0.465, 0.477) to red‐orange at 77 K (0.570, 0.420). Although these planar Ag(I) complex phases exhibit good reversibility and stability, the fluorescence transition primarily occurs below room temperature (77 K), which ultimately limits their application in many real‐life scenarios.

**FIGURE 13:**
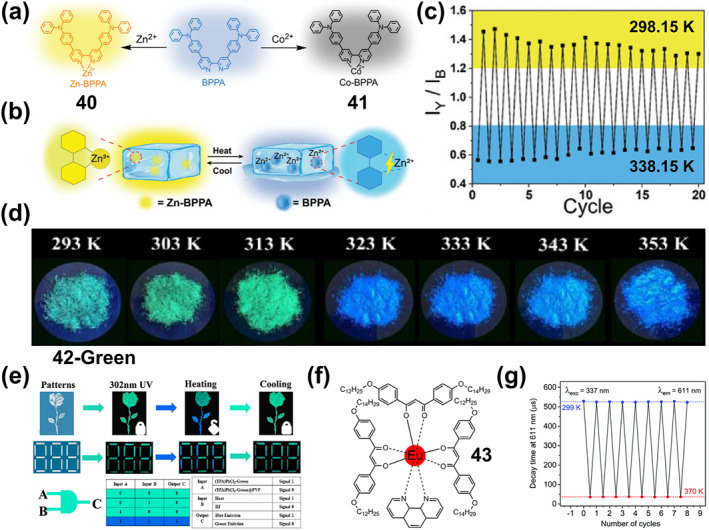
(a) Chemical structures of BPPA (blue emission), **40** (yellow emission) and **41** (no emission). (b) Schematic illustration describing the thermochromic luminescence induced by reversible coordination and dissociation of metal‐ligand (M‐L) interactions. (c) The 20 cycles of intensity ratio variations measured at 298.15–338.15 K; Reproduced with permission: copyright 2020, John Wiley and Sons.^[103c]^ (d) Luminescence images (under 302 nm UV lamp) of **42‐Green** after heating at different temperature. (e) Fluorescent pattern flower and digital message for message encryption and schematic diagram of optical logic and gates based on complexes **42‐Green** and **42‐Green@PVP**. Reproduced with permission: copyright 2022, American Chemical Society.^[110]^ (f) The chemical structure of complex **43** and (g) its reversible changes in the luminescence decay time at *λ*_em_ = 611 nm in consecutive heating–cooling cycles between 299 and 370 K (right). Reproduced with permission: copyright 2020, Royal Society of Chemistry.^[106c]^

In order to realize thermochromic materials with simultaneously reversible and lower transition temperatures, Ma et al. prepared metal complexes (**40** and **41**) (Figure 13a) formed from Zn^2+^ or Co^2+^ with 4,4′‐([2,2′‐bipyridine]‐4,4′‐diyl)bis(N,N‐diphenylaniline) (BPPA) ligands and introduced them into a PEG matrix to construct chameleon‐like TLMs.^[103c]^ Such TLMs work by reversible metal coordination or dissociation between Zn^2+^ and BPPA ligands or by excited state conformational changes. When the temperature increases, the motion of the polymer chain increases, causing dissociation of the metal coordination bond and the complex exhibits blue fluorescence. When the temperature was decreased, Zn^2+^ formed complexes with BPPA again and the complexes **40** showed yellow color (Figure 13b). The complexes **40** exhibited good reversibility and were recorded 20 times repeatedly at 298.15 and 338.15 K without any detectable intensity change (Figure 13c). Similarly, thermochromic materials with excellent cycling stability and relatively low transition temperatures were recently prepared from a new one‐dimensional (1D) organic‐inorganic lead chloride hybrid single crystal **42‐Green** (namely, green‐emitting crystals, (TPA)PbCl_3_‐Green (TPA = tetrapropylammonium)) by Zhang et al.^[110]^ As shown in Figure 13d, **42‐Green** crystals changed the emitted light from green to blue under 302 nm excitation after heating at 323 K for 30 min, indicating that **42‐Green** crystal transformed to **42‐Blue** (i.e., (TPA)PbCl_3_‐Blue crystals showing blue emission) with sensitive thermochromic phenomenon. After exposure of **42‐Blue** to room temperature air, the initial fluorescence was restored after natural cooling to room temperature over the course of about 660 s. By comparing the changes in bond lengths and bond angles of [Pb‐Cl6] octahedra in **42‐Green** and **42‐Blue**, the authors found that under thermal stimulation, the structures of the ground state **42‐Green** and **42‐Blue** octahedra deform to different degrees, which may lead to the excited‐state structures differently, resulting in different Stokes shifts. Due to the increase in temperature, the self‐trapped exciton can acquire enough thermal energy to overcome the energy barrier caused by the two triplet excited states. Further, they realized dynamic anti‐counterfeiting and message encryption as well as optical logic gate applications using **42‐Green@PVP** ((TPA)PbCl_3_‐Green@PVP complexes, PVP = poly(vinylpyrrolidone)) as a fluorescent security ink. As shown in Figure 13e, both **42‐Green** and **42‐Green@PVP** display incorrect patterns and numbers under 302 nm UV irradiation and emit green luminescence, effectively creating an encrypted locked state. **42‐Green** covered patterns and digits become blue emitting after the use of a heat‐treated unlocking key, allowing the true encrypted information to be read. After cooling at room temperature, the blue emission reverts to green, again shielding the message and ensuring cryptographic security.

In order to meet the needs of practical applications, in addition to the reversible response at lower temperatures, improving the response temperature range and sensitivity of thermochromic fluorescent materials is also an important direction to investigate. To achieve this goal, Lapaev's group reported a new asymmetric europium(III) *β*‐diketonate complex powder **43** (Figure 13f), and prepared temperature‐responsive films as a novel temperature probe.^[106c]^ The films exhibit bright Eu^3+^ ion luminescence due to the efficient energy transfer from the ligand to the metal ions. Continuous cooling and heating cycling tests in the range of 270–370 K revealed that the film exhibited a fully reversible temperature‐dependent change in the luminescence decay time with an average absolute sensitivity of −5.4 μs.K^−1^ (Figure 13g). Also, the film has the advantages of luminescence insensitivity to oxygen, complete UV resistance and excitation by light sources in a wide wavelength range (280–425 nm), making it a promising reusable luminescence thermometer.

### 4.3 Thermal‐magnetic responsive bistable coordination complexes

**FIGURE 14:**
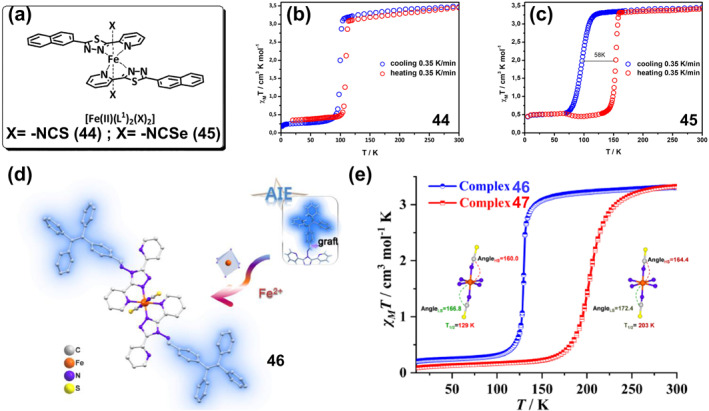
(a) Chemical structures of **44** and **45**; magnetic susceptibilities (*χ*_M_ *T*) of (b) **44** and (c) **45**; Reproduced with permission: copyright 2022, American Chemical Society.^[113]^ (d) The structural design of **46** at the molecular level. (e) Magnetic susceptibilities (*χ*_M_ *T*) of **46** and **47** as a function of temperature (*T*). Reproduced with permission: copyright 2023, Elsevier.^[114]^

Temperature‐responsive spin‐crossover (SCO) compounds, which trigger spin switches upon thermal stimulation, are bistable smart materials for building temperature‐responsive molecular switches, molecular memory devices, and sensors.^[111]^ For practical applications, to develop SCO transitions with a wide thermal hysteresis, that is, significantly different critical temperatures for the cooling and heating modes around room temperature is a prerequisite as it provides the desired memory effect.^[112]^ For this reason, Kiehl et al. report two isostructural complexes (**44**–**45**) (Figure 14a) [Fe^II^(L^npdtz^)_2_(NCX)_2_] (X = S; Se) of the bifurcated chelating ligand Lnpdtz (2‐naphthyl‐5‐pyridyl‐1,2,4‐thiadiazole), which achieved a wide range of thermally hysteresis spin‐crossover (SCO) effects and the coexistence of thermally induced metastable HS states at low temperatures.^[113]^ The complex **44** showed very abrupt spin transitions at 100 K during cooling and at 110 K during heating, resulting in a hysteresis of 10 K (Figure 14b). **45** shows an abrupt spin transition at 154 K during the heating process and at 96 K during the cooling process, resulting in a hysteresis of 58 K (Figure 14c). At room temperature, **44** and **45** are in the HS state with magnetic susceptibilities (*χ*_M_ *T*) values of 3.47 and 3.46 cm^3^ K mol^−1^ at 300 K. Among them, the overall hysteresis Δ*T*_1/2_ of **44** is more than 16 K.

Single spin transition, however, is often very limiting for the application of SCO materials. Therefore, through the continuous breakthroughs, SCO materials have been prepared, which have a variety of physical properties, such as electrical conductivity, nano‐magnetism, liquid crystals, and nonlinear optical (NLO) properties, leading to the integration of multiple functions in a single material. In particular, bifunctional luminescent SCO materials have received significant attention because they can track fluorescent signal modulation through spin‐state switching processes. However, the construction of luminescent SCO compounds is difficult because transition metal ions (especially Fe(II)) often cause fluorescence quenching in the solid‐state. To solve this problem, Li's team has reported a new method to construct luminescent SCO compounds by introducing an aggregation‐induced emission (AIE) fluorophore (or AIEgen), showing a synergistic effect between SCO and solid‐state fluorescence.^[114]^ They first prepared a novel ligand N‐(3,5‐bis(pyridin‐2‐yl)‐4H‐1,2,4‐triazol‐4‐yl)‐1‐(4‐(1,2,2‐triphenylethenyl)phenyl)methanimine (abpt‐TPE) containing AIE luminescent groups (i.e., tetraphenylethenylene and coordinated it with two isothiocyanato anions and Fe(II) to obtain two bidentate luminescent SCO complexes were obtained as [Fe(abpt‐TPE)_2_(NCS)_2_]‐4MeCN (**46**) (Figure 14d) and [Fe(abpt‐TPE)_2_(NCS)_2_]‐2MeCN‐2CH_2_Cl_2_ (**47**). Both **46** and **47** exhibit one‐step and abrupt SCO behavior, with a transition temperature (*T*_1/2_) of 129 K for compound **46** and 203 K for compound **47**, and a difference of nearly 74 K between the two *T*_1/2_ values(Figure 14e). There is some difference in the magnetic data of the two complexes as the temperature changes. For compound **46**, the *χ*_M_ *T* value of Fe^II^_LS_ increases from 0.21 cm^3^ K·mol^−1^ at 10 K to 3.24 cm^3^ K·mol^−1^ at 300 K, corresponding to Fe^II^_HS_. For compound **47**, the *χ*_M_ *T* value increases from 0.08 cm^3^ K·mol^−1^ at 10 K to 3.30 cm^3^ K·mol^−1^ at 300 K (consistent with the presence of Fe^II^_HS_). Through a comprehensive examination of solid‐state fluorescence, the emission wavelength of **46** was identified as *λ*_max_ = 465 nm under room temperature excitation at 365 nm, manifesting a blueshift of 13 nm relative to the ligand's emission wavelength (abpt‐TPE, *λ*_max_ = 478 nm). This suggests that the fluorescence of abpt‐TPE was successfully maintained after the chelation process with Fe(II) ions. The authors investigated the correlation between the SCO phenomenon and the fluorescence behavior of both the ligand and the complexes through variable temperature fluorescence emission spectroscopy. Within the temperature range of 80–300 K, the abpt‐TPE ligand displayed a solitary peak at 478 nm. The emission intensity exhibited a gradual decline with increasing temperature, indicating the presence of thermal fluorescence bursts. In contrast, both **46** and **47** demonstrated significantly different fluorescence behaviors. Compound **46** exhibited two emission peaks at 100 K, with *λ*_max_ values of 441 and 465 nm. The relative intensities of these peaks gradually increased with temperature within the 100–220 K range. This observation aligns well with the magnetization data for **46**, given the LS to HS transition occurring between 100 and 220 K. Beyond 220 K, fluorescence intensity declined due to thermal quenching, consistent with magnetization data indicating the persistence of the Fe^II^_HS_ state above 300 K. In contrast, the intensity of the peak at *λ* = 457 nm for complex **47** decreases as the temperature rises from 80 to 190 K. However, upon further heating to 320 K, the emission intensity not only increases but also undergoes a redshift of 17 nm (*λ* = 474 nm), reaching its maximum at 320 K, approximately six times higher than that at 80 K. The fluorescence intensity at *λ* = 457 nm also diminishes between 80 and 190 K. The variation in fluorescence intensity witnessed from 190 to 320 K corresponds to the magnetic behavior of compound **47**. This suggests that spin‐state‐dependent fluorescence emission was observed throughout the entire temperature range, with LS state fluorescence quenching and HS state fluorescence enhancement. Time‐dependent DFT computations reveal that the most prominent absorption bands in the LS state align with the π →π* transitions of the abpt‐TPE ligand coupled with a partial MLCT component. The two weak absorption bands in the higher wavelength region (529 and 705 nm) originate from the d(Fe) + π(SCN)→π*(abpt‐TPE) transitions attributed to the MLCT. The HS state of complex **46** primarily arises from the π→π* transitions within the ligand, while complex **46** is associated with the d(Fe) + π(SCN)→π*(abpt‐TPE) transitions. The most intense absorption band in the HS state experiences a blueshift of approximately 15 nm in comparison to the LS state. Consequently, luminescence intensifies during the spin transition from the LS state to the HS state, with the fluorescence intensity of the abpt‐TPE ligand contingent upon HS‐LS.

### 4.4 Thermally triggered single crystal to single crystal phase transition in bistable coordination complexes

Thermally triggered SCSC phase transition materials are excellent candidates for constructing bistable smart materials. Temperature‐triggered SCSC solid‐state transitions originate from two basic mechanisms: the release of solvent molecules regulated by temperature,[^82^, ^115^] and the temperature‐induced structural changes resulting in polymerization or depolymerization, leading to bond breaking/formation and the promotion of lattice motion.^[116]^

**FIGURE 15:**
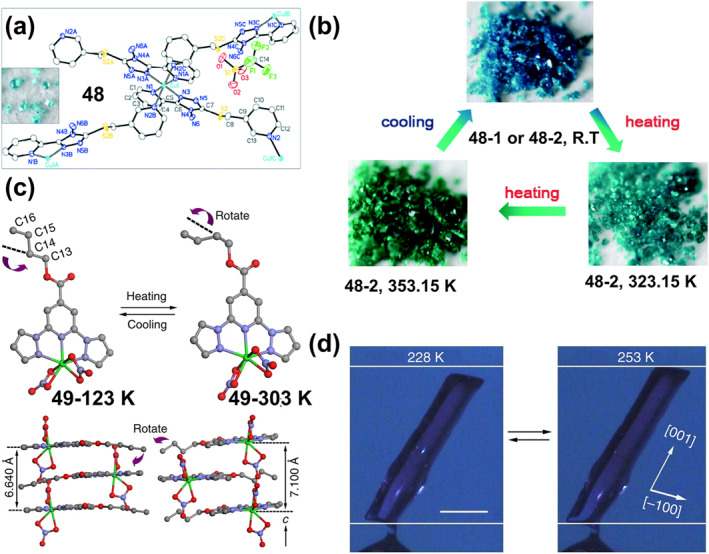
(a) Crystal structure of **48**, (b) the color change of **48** in the solid state with temperature change. Reproduced with permission: copyright 2014, Royal Society of Chemistry.^[116e]^ (c) Molecular structures of crystal **49** and the n‐butyl group of the ligand rotates ca. 100° around the C13–C14 bond following phase transition. Green, Co; gray, C; blue, N; red, O. (d) The crystal length changes from 1.78 to 1.90 mm along [001] from 228 to 253 K and switches back to the original state upon cooling, scale bar represents 500 μm; Reproduced with permission: copyright 2015, Springer Nature.^[117]^

Although the temperature‐responsive reversible SCSC process has been widely reported, it is still difficult to observe this transformation process directly. Dong's group first visualized the single‐crystal to single‐crystal transition by preparing a reversible SCSC complex with thermochromic properties.^[116e]^ This SCSC thermochromic complex **48** is a novel CPs based on triazole‐bridged asymmetric organic ligands (Figure 15a). The complexes show good thermal stability and interesting thermochromic properties while undergoing the SCSC transition process. In the solid state, the crystals **48** are sky blue at room temperature, which change to green when the temperature is increased to 353.15 K (Figure 15b). Once the temperature is restored to room temperature, the color is restored accordingly. These crystals with different colors were shown to all crystallize in the monoclinic space group of *P*2(1)/n and have the same stacking pattern in the solid state.

This macroscopic visualization of color changes achieved by molecular level transformations facilitates the detection of SCSC processes and, in turn, grants macroscopic materials a wider range of applications. Therefore, the amplification of this developed reversible molecular level motion to macroscopic states obtained through a synergistic effect is of great significance but extremely challenging due to the subtleties of molecular motion in solids, especially crystals. For this purpose, Su et al. constructed crystalline cobalt(II) complexes with thermally induced SCSC using rotationally isomerized molecular motions around carbon‐carbon bonds to achieve the conversion of alkyl rotations into macroscopic crystalline reversible macroscopic motions.^[117]^ As shown in the Figure 15c, they synthesized prismatic purple crystalline state cobalt(II) complexes **49** [Co(NO_3_)_2_(L)] (L = n‐butyl‐2,6‐di(1H‐pyrazol‐1‐yl) containing n‐butyl groups in the ligand, where the n‐butyl group acts as a potential rotor, thus allowing the complex to exhibit reversible crystalline deformation at the temperature of structural phase transition. The n‐butyl group of the ligand freezes in the low‐temperature phase, but when being heated to 253 K, it rotates around the C‐C bond by an angle of about 100° during the phase transition, resulting in an increase in crystal size of 6%–7% along the molecular stacking direction. The crystal length changes from 1.78 to 1.90 mm along [001] from 228 to 253 K and switches back to the original state upon cooling (Figure 15d). More importantly, this transition can be cycled many times without causing crystal deformation.

## 5 ELECTROCHEMICALLY RESPONSIVE SYSTEMS

Similar to light stimulation, electricity is one of the most readily available energy sources with the advantages of simplicity of operation, accessibility, precision control, non‐contact, rapid and reversible induced transitions, and stable energy output.^[118]^ It is particularly worth noting that the signal size can be tuned and easily made compatible with conventional batteries and electrode materials,^[119]^ enabling integrated intelligent operation in combination with other devices such as capacitors and sensors.^[120]^ Due to the existence of some unique properties of metal centers and ligands, such as structural transformations, redox state changes, and reversible transfer of protons, more and more electrical stimulation‐responsive materials based on metal complexes are being developed.[^93^, ^121^] Moreover, metal complex‐based electroresponsive materials have richer properties and higher stability than those based on inorganic transition metal oxides or organic small molecules or polymers.^[122]^ Some redox‐driven metal complexes and organometallic compounds, which undergo structural rearrangements in response to electrochemical stimuli, have a pronounced bistability effect. This bistability of the complex requires that the two redox states of the molecule alternate within the electrochemical potential range but can reversibly interconvert at potentials outside the bistable interval.[^7^, ^123^] Herein, several important electrostimuli‐responsive metal complexes are presented, including EC complexes, electrofluorescent complexes, or molecular magnetic switches.

### 5.1 Electrochromic bistable coordination complexes

**FIGURE 16:**
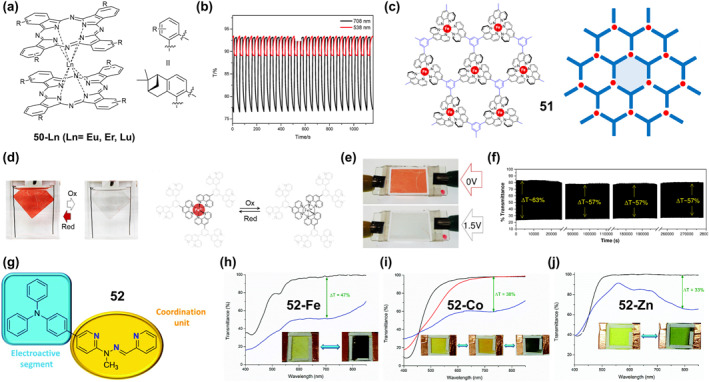
(a) Structure of **50‐Ln** (Ln = Eu, Er, Lu), (b) *T*% changes of **50‐Eu–ITO** at two wavelengths versus time by repeating the potential steps using 0 V and +1.0 V. Reproduced with permission: copyright 2015, Royal Society of Chemistry.^[134]^ (c) the chemical structure of complex **51** and its schematic representation of hexagonal coordination nanosheets. (d) Electrochromic (EC) change of nanosheets from intense red to completely transparent, and its chemical changes during redox transformation. (e) Reversible color change of the device from intense red to completely transparent. (f) Long‐term cycling stability of the device exceeds 15,000 cycles. Reproduced with permission: copyright 2020, American Chemical Society.^[135]^ (g) Schematic representation of ligand **52**. Transmittance spectra of the EC device containing the complexes (h) **52‐Fe**, (i) **52‐Co**, and (j) **52‐Zn** as an active material and (inset image) their photographs of the device in different redox states. Reproduced with permission: copyright 2020, Royal Society of Chemistry.^[136]^

Electrochromism (EC) is a phenomenon involving a change in color in response to an applied voltage.^[124]^ Bistable EC materials and devices are of great interest because they can reversibly switch between two stable optical states under electrical stimulation.^[125]^ Bistable EC materials, which are characterized as absorption memory effect, have the advantages of high energy efficiency, ultra‐low energy consumption[^12^, ^126^] and long‐term maintenance of the desired color/transmittance because no additional energy consumption is required to maintain the desired optical state.^[127]^ Thus, electrochromic devices (ECDs) based on bistable EC materials show significant potential as a future energy‐efficient optoelectronic technology for smart windows,[^12^, ^126^, ^128^] recordable information displays,^[129]^ e‐paper,^[130]^ optical communication and visual energy storage.^[131]^ The metal complexes or CPs, which undergo color change by MLCT mechanism,[^130^, ^132^] integrate the advantages of organic and inorganic materials and are promising alternative solutions for the development of bistable EC devices with low energy consumption. As a result, significant progress has been made in metal coordination‐based EC materials with excellent color rendering efficiency and redox stability.[^126^, ^133^] However, most of the EC materials exhibit EC behavior in the visible or near‐infrared (NIR) region, and there are still difficulties in expanding the wavelength response range for EC devices to adapt to various complex environments. To achieve this, in 2015, our group prepared an octa‐pinene‐substituted bilayer lanthanide(III) phthalocyanine (Figure 16a), **50‐Ln** (Ln = Eu, Er, Lu), which exhibits EC behavior in both the UV‐visible and NIR regions, by introducing bulky and hard pinene moieties into the phthalocyanine.^[134]^ The increased intramolecular distance between the macrocycles in this bilayer lanthanide(III) phthalocyanine leads to a significant redshift of the ring‐to‐ring valence CT band. In particular, the intervalent bands of **50‐Eu**, **50‐Er**, and **50‐Lu** are at 1944, 1693, and 1620 nm, respectively. Due to the NIR absorption redshift of the complexes and having different oxidation states and colors, the **50‐Ln** solutions exhibited EC behavior in both UV‐visible and NIR regions. Among them, the spectral variation of **50‐Eu** covers almost the entire NIR region. Due to the introduction of pinene moiety to improve the solubility of bilayer lanthanide (III) phthalocyanine complexes, we prepared EC devices **50‐Ln‐ITO** by spin‐coating a thin film of **50‐Ln** complex with good film‐forming ability onto ITO‐coated glass. In the absence of an external potential effect, **50‐Ln‐ITO** shows green color. After the anodic potential process, the color of the film changed to pink due to the oxidation of **50‐Ln**. During the reverse CV scanning process, its color returns to green again as **50‐Ln** changed from the oxidized state to the reduced state. The prepared devices have reversible EC performance with high color rendering efficiency and good stability, as shown in Figure 16b, and the optical contrast (*T*%) can be detected only a small change in multiple cycles.

While EC materials demonstrate effective controlled dynamic color changes when subjected to suitable electrical stimulation, the development of EC devices with superior optical contrast, extended durability, and lower operating voltage presents a critical challenge in this domain.[^121^, ^132^, ^133^] In this context, Higuchi's team synthesized double‐branched dense hexagonal nanosheets (complex **51**) based on Fe(II) coordination (depicted in Figure 16c) and effectively fabricated EC devices through the implementation of the liquid/liquid interfacial approach to tackle these challenges.^[135]^ They first assessed the EC properties of the nanosheet films in a non‐aqueous solution. Throughout EC assessment, the films displayed reversible transitions in coloration, shifting from deep red to colorless, and vice versa, upon the application of potentials at +1.0 and 0 V, as illustrated in Figure 16d. The alteration in color was linked to a reversible oxidation/reduction process involving the conversion of Fe(II) to Fe(III) and its reverse for the central metal ion within the nanosheets (Figure 16d, right). The dynamic transmittance switching between colored and bleached states was observed at *λ*_max_ = 518 nm through the application of constant operating voltages at +1.0 and 0 V, as depicted in Figure 5e. The findings indicate rapid state transitions on a 1 × 1.2 cm^2^ active area nanosheet film: bleaching time (*t*_*b*_) = 3.3 s and coloring time (*t*_*c*_) = 2.9 s, emphasizing the swift coloration kinetics of the nanosheets. Additionally, they developed ECDs using this complex **51** as the EC layer. The devices transitioned from a dark red to colorless at a voltage of 1.5 V. Subsequent application of a 0 V operating voltage resulted in the reappearance of the deep red color (Figure 16e). Furthermore, the durability of the device was assessed by continuously tracking the optical contrast (Δ*T*) over time under an operating voltage of +1.5/0 V with a 15 s hold time. The result showed that the device exhibited exceptional cycling stability, demonstrating minimal variation in Δ*T* over 15,000 cycles (Figure 16f). This material achieved remarkable durability along with substantial contrast and recurring color alterations under low applied operating voltages.

An essential consideration in the design of EC materials is the evident contrast in color between the two redox states. Nevertheless, a significant proportion of research has concentrated on the transition from colored to transparent,^[137]^ with fewer reports on materials transitioning from transparent to dark or even black.^[138]^ The primary reasons for this phenomenon can be attributed to two main factors: 1) the complexity involved in acquiring a material that effectively absorbs within the visible spectrum (400–750 nm) in its colored state and completely fades within the same range in its bleached state, and 2) based on the theory of mixing colors, the difficulty in obtaining dark‐colored materials primarily stems from their non‐primary colors. For instance, the realization of green and brown colors in EC compounds is a challenge. This is because that the production of the green color necessitates the absorption of at least two chromophores in the yellow and blue regions of the visible spectrum, and these absorption bands have to be simultaneously controlled by an applied potential. Similarly, achieving the brown color involves the intricate task of combining the colors of two dyes, such as red and green, orange and blue, or yellow and violet.^[139]^ To address these issues, Banasz et al. synthesized three innovative complexes incorporating the hydrazone N3‐donor ligand **52** (Figure 16g) through coordination with metal ions, specifically, the Fe(II) complex **52‐Fe**, the Co(II) complex **52‐Co**, and the Zn(II) complex **52‐Zn**.^[136]^ The resulting materials demonstrated the capability to produce green and brown colors within the EC device. As shown in Figure 16h, the device incorporating **52‐Fe** exhibits increased transmittance in the blue region when in a neutral state, leading to the manifestation of a high‐transmittance yellow color. Upon application of a +2.0 V voltage, the transmittance decreases to approximately 51% at 705 nm, thereby yielding a color contrast of about 47% for the device. In contrast, complex **52‐Co** demonstrated the presence of three colors corresponding to three distinct redox states. Under a potential of +1.3 V, the device transitions from yellow to orange, attributed to the oxidation of the central metal ion and the development of the complex **52‐Co**. Subsequently, under a potential of +2.0 V, the device experiences a secondary change in color from orange to brown‐green, attributed to the oxidation of the TPA group and representing a discoloration of the ligand group (Figure 16i). Meanwhile, the color of the complex **52‐Zn** shifts from yellow to green, with the green color stemming from the amalgamation of the MLCT band in the yellow region and the broad absorption band at 794 nm (Figure 16j). Additionally, the complexes exhibit favorable EC stability. By monitoring the transmittance of the complexes **52‐Fe**, **52‐Co**, and **52‐Zn** at 705 nm, 730 and 790 nm with time, it was revealed that transmittance variations of 47.5%, 37.2%, and 33.7%, respectively, following 140 cycles of switching tests between fully oxidized and reduced states. These EC materials that transition from a dark to a transmissive state hold potential applicability across various display devices such as digital signage, e‐paper, as well as future applications in smart windows or EC sunglasses.

### 5.2 Electrofluorochromic bistable coordination complexes

Electrofluorochromic (EFC) materials are a special type of electroluminescent (ELC) material which exhibits reversible changes in emission properties and color under electrical stimulation.[^93^, ^140^] Functional molecules, metal complexes, and polymer films based on EFC systems undergo redox state change under electrical stimulation^[140d]^ and are able to achieve reversible modulation of fluorescence intensity and color simultaneously,[^140^, ^141^] hence they are promising materials for building dual‐mode operations in emission and reflection.^[142]^ Metal‐organic complex‐based EFC materials have attracted attention due to their advantageous longer lifetime than organic systems, rich excited state properties, good redox reversibility, high emission quantum yields, and exhibit multicolor tunable emission over a broad wavelength region.^[143]^ These metal complexes usually contain metal centers with d^6^, d^8^, and d^10^ configurations (e.g., Ir^III^, Ru^II^, and Pt^II^) or lanthanides.^[143b]^ The fundamental mechanism of electrofluorochromism usually involves redox reactions in the fluorophore itself with electrochemical switching properties or quenching of the luminescence of the redox‐inactive fluorophore by electron or energy transfer from the external redox‐active switching moiety.^[144]^ In this context, the redox‐controllable luminescent cyclometalated dinuclear Pt^II^ complexes have emerged as attractive functional materials for the manufacture of OLED due to the excellent luminescent properties of the polynuclear Pt^II^ complexes and the abundant redox behavior resulting from electronic Pt‐Pt interactions.^[145]^

**FIGURE 17:**
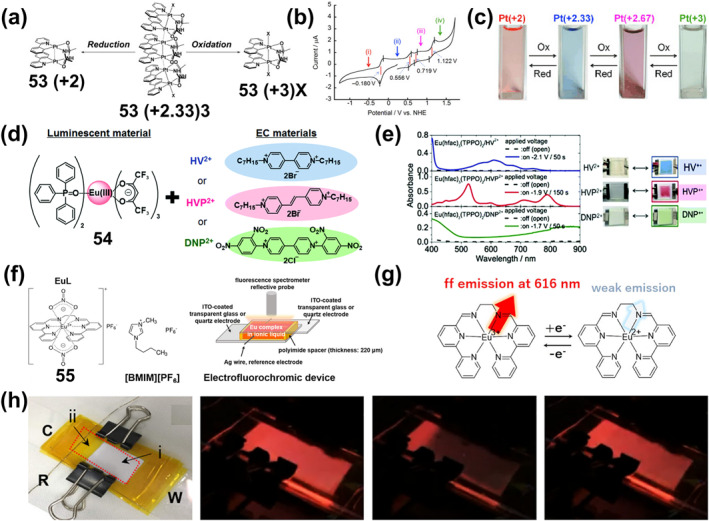
(a) Redox reactions of complex **53**. (b) Cyclic voltammogram of **53** under N_2_ atmosphere, recorded at a scan rate of 5 mV s^−1^. (c) Photographs of DMF solutions of **53** in each oxidation state. Reproduced with permission: copyright 2015, John Wiley and Sons.^[146]^ (d) Structures of luminescent moiety **54** and electrochromic (EC) molecules of electrochemical photoluminescent (PL) multicolor systems. (e) Absorption spectra of the two‐electrode cell containing EC complex solutions (left) and the coloration changes induced by the electrochemical reaction under visible light irradiation. Reproduced with permission: copyright 2017, Royal Society of Chemistry.^[147]^ (f) Molecular structures of complex **55** and the ionic liquid [BMIM][PF6], and the scheme of electrofluorochromic device, (g) the EC mechanism, (h) Image of a switching device under room light and images of the device under a 365 nm handheld UV lamp during the fluorescence switching at different potentials applying 5 s each. Reproduced with permission: copyright 2020, American Chemical Society.^[140c]^

However, since OLED displays images that are difficult to see in direct sunlight and ECDs cannot be used in the dark, these display devices have limited visibility in practical applications. To overcome these drawbacks, the development of dual electrochromic/electroluminescent (EC/EL) display devices is extremely critical. To achieve this goal, Yoshida et al. reported the first redox‐multistable dinuclear motif platinum complex **53** for electrochemically controlled electric (*E*) field strength multicolor electrochromism and luminescence.^[146]^ The obtained complexes exhibit multi‐step redox states (Pt(+3), Pt(+2.67), Pt(+2.33), Pt(+2.5) and Pt(+2)) (Figure 17a), in which the bivalent complexes exhibit intense red phosphorescence, the trivalent complexes are non‐fluorescent, and the red‐emitting Pt^II^‐Pt^II^ and non‐emitting Pt^III^‐Pt^III^ states can be reversibly controlled through electrical stimulation. As shown in Figure 17b, the CV measurements of **53** clearly show four redox pairs with *E*_1/2_ = −0.180, 0.556, 0.719, and 1.122 V relative to Normal Hydrogen Electrode. The three redox pairs observed at the anode side were rationally assigned to the Pt(+2.33) state, which is progressively oxidized to Pt(+2.5), Pt(+2.67) and finally to the trivalent state Pt(+3). In contrast to the redox behavior, **53** exhibits remarkable polychromatic EC behavior, whereby color changes from blue to pink, yellow and orange can be observed by applying different voltages (Figure 17c).

In addition to the above‐mentioned multicolor steady‐state electrochemical fluorescence and electrochromism induced by valence changes, the complexation of europium(III) complexes as luminescent molecules with different chemically active molecules is also an effective strategy to realize the electrochemical switching between absorption and luminescence in multi‐functional systems.^[148]^ For example, in 2017, Kobayashi's team. reported and synthesized electrochemical PL multicolor systems based on cyan‐ocean‐green (CMG) viologen components by combining luminescent Eu^3+^ chelates with viologen derivatives.^[147]^ The system mainly includes the luminescent molecules tris(hexafluoroacetylacetatol)europium(III) bis(triphenylphosphine oxide), **54** [Eu(hfac)_3_(TPPO)_2_], and electrochemically active color‐changing quenchers, respectively, 1,1′‐diheptyl‐4,4′‐bipyridinium dibromide (HV^2+^, from transparent to cyan), 1,1′‐diheptyl‐4,4′‐vinylene bipyridinium dibromide (HVP^2+^, from transparent to magenta), 1,1′‐bis(2,4‐dinitrophenyl)‐4,4′‐bipyridinium dichloride (DNP^2+^, from clear to green) (Figure 17d). Among them, the luminescent moiety **54** is the redox‐inactive part, while HV^2+^, HVP^2+^, and DNP^2+^ are used as external redox‐active switching moieties to modulate the luminescence of the Eu^3+^ chelate through the FRET mechanism under electrical stimulation. **54** exhibits intense red photoluminescence when the viologen derivatives are present in the colorless state, and after electrochemical reduction of the generated colored viologen derivatives, the Eu(III) complexes luminescence is rapidly quenched (Figure 17e).

Nevertheless, these typical electrofluorescent color changes are often achieved by controlling the redox state of ligands or other electroactive species for energy transfer to the fluorophores, which is not conducive to practical and long‐term applications. To overcome these problems, Kim et al. reported a strategy to realize the single fragment as both a photoemitter and a redox switch using a complex (EuL) with a central europium (Eu) ion and a hexadentate pyrimidine derivative.^[140c]^ They prepared three‐electrode electrochemical switching devices using the ionic liquid [BMIM][PF_6_] and EuL, where the reversible electrochemical reaction of the complex **55** between Eu(III) and Eu(II) produced a large emission modulation and luminescence in the emitting and quenched states, respectively (Figure 17f). Due to the stable helical coordination structure of the ligand in [BMIM][PF_6_], only a small amount (≤1 wt%) of EuL is required to significantly enhance the red fluorescence. The electrofluorescence photochromic device achieves a large and fast contrasting luminescence response as the emission is sharply quenched in the reduced state (Eu^2+^) yet successfully recovered by subsequent oxidation (Eu^3+^) (Figure 17g). Reversible fluctuations in the excitation spectrum and emission spectrum of the fluorescence photochromic device are achieved within a potential window of ±2 V (Figure 17h). This design strategy opens up an efficient and intelligent pathway for Eu luminescence control in optoelectronic devices.

### 5.3 Electrochemically responsive molecular magnetic switch based on bistable coordination complexes

**FIGURE 18:**
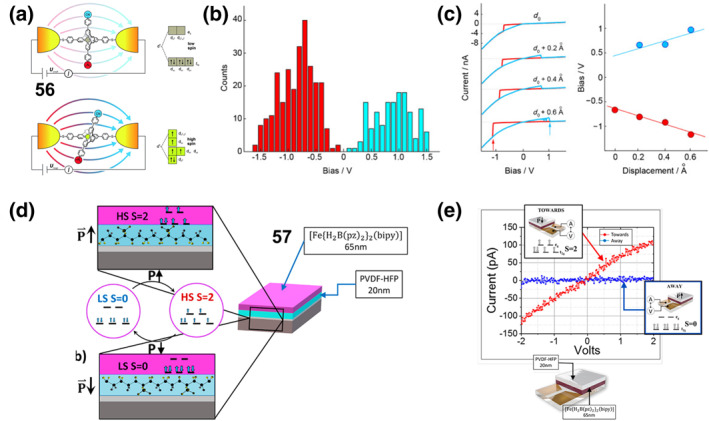
(a) Idealized sketch of the voltage‐triggered spin crossover (SCO) switch in a single‐molecule junction. Top: Low‐spin Fe(II) complex **56** bridging the two electrodes at a small applied voltage. Bottom: High‐spin Fe(II) complex **56** with distorted coordination sphere due to the alignment of the push‐pull system in the applied electric field; (b) Histogram of the switching bias voltage for all *I‐V* plots of **56** displaying bistability features. (c) Current‐voltage characteristics for increasing electrode separation showing a hysteretic switching behavior (left) and Switching bias voltage as a function of electrode separation for the *I‐V* data (right). Reproduced with permission: copyright 2015, John Wiley and Sons.^[152]^ (d) The spin state of the **57** (red) when coupled to the polarization of a ferroelectric PVDF‐HFP layer (blue). (e) The changes in conductance observed via polarizing the ferroelectric toward complex **57** in different ferroelectric polarization directions. Reproduced with permission: copyright 2020, American Chemical Society.^[153]^

As mentioned above, spin‐crossover metal complexes exhibit stimulus‐responsive switching and bistable properties at the molecular level making them excellent candidates for the development of smart bistable magnetic molecular materials. In particular, electrical stimulation, usually in the form of currents, voltages, and electric fields, reversibly switches the spin‐crossover metal complexes in an effective, direct, and contact‐free manner.^[149]^ Interestingly, the electric field can be used directly to manipulate the single nuclear spin resonance, so that spin and electric field are not directly coupled in the reversible conductance switching process.^[150]^ In this context, spin cross complexes are integrated as functional components in nanocrystals, nanoparticle arrays, multilayer structures, and bulk materials, and have been studied in surfaces of various concentrations ranging from submonolayers to thin films.^[151]^ However, the reversible electrical stimulation response of SCO compounds at the single‐molecule level remains challenging even it is responsive to the applied voltage or current. To this end, in 2015, Mayor's group reported a single‐molecule switch concept based on terpyridine iron(ii) ligand sphere‐dependent spin states (Figure 18a).^[152]^ In this system, one ligand acts as a bridge by securing the complex **56** in the junction, and the other ligand exhibits an intrinsic dipole moment in response to the applied electric (*E*) field strength. At the threshold voltage, the dipole moment senses the electric field and causes the distortion of the Fe^II^ coordination sphere. The central Fe^II^ ion changes from a LS state to a HS state, resulting in bistability of the current‐voltage curve (Figure 18b,c).

In addition to using electric fields to control spin states, voltage‐controlled molecular multiferroics provide an exciting new platform for exploring non‐volatile spin‐state switching, providing the basis for switchable nanoscale molecular devices with spin‐state bi‐stability. For instance, Mosey et al. achieved voltage‐controlled molecular nonvolatile spin‐state switching by loading complex **57** Fe{H_2_B(pz)_2_}_2_(bipy)] (pz = tris(pyrazol‐1‐1y)‐borohydride, bipy = 2,2′‐bipyridine) on molecular ferroelectric poly(vinylidene fluoride hexafluoropropylene) (PVDF‐HFP) substrates (Figure 18d).^[153]^ As in Figure 18e, when the ferroelectric PVDF‐HFP is polarized upward, complex **57** tends to the HS state with *S* = 2 and yields a large conductance. When the ferroelectric PVDF‐HFP is polarized downward, it leads to a low‐spin (*S* = 0) state with a decrease in conductivity by two orders of magnitude. However, for 65 nm thick films of **57**, the spin state is not only dependent on the direction of ferroelectric polarization but also on the temperature. In the presence of voltage, such films can switch between spin and low‐spin states at room temperature with a significant change in conductivity. In the absence of external electrical stimulation, complex **57** is naturally in the HS state at 298 K, and the typical T_1/2_ for going from the HS state to the LS state is about 167 K.

**FIGURE 19:**
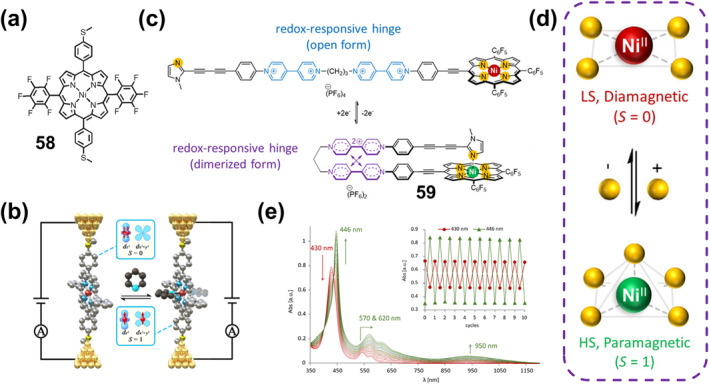
(a) Chemical structure of **58**. (b) Reversible coordination reaction between **58** and 3,5‐lutidine in solution, and the related spin switch (*S* = 0 ↔ *S* = 1). Reproduced with permission: copyright 2021, Chinese Chemical Society Publishing.^[155]^ (c) Electron‐triggered reversible folding/unfolding process in **59** accompanied by coordination/dissociation of the endogenous ligand and (d) its spin‐state switching by coordination/dissociation of an exogenous ligand to/from a Ni (II)‐porphyrin; (e) UV/vis spectra recorded during the exhaustive electrolysis of **59**. Increment: Switching cycles of **59** on thin‐layer spectroelectrochemistry during alternating reduction (Eapp = −0.85 V) and reoxidation (Eapp = 0 V). Reproduced with permission: copyright 2022, American Chemical Society.^[156]^

Most of the spin switches, with low spins stabilized at low temperatures and high spins stabilized at high temperatures, require low‐temperature vacuum conditions,^[154]^ so increasing the operating temperature to room temperature is an important challenge to overcome. To address this problem, Li et al. designed a room‐temperature spin‐state single‐molecule conductivity switch via an coordination‐induced spin‐state switching approach, using the square planar nickel(II) porphyrin derivative, 5,15‐bis(4‐(methylthio)phenyl)‐10,20‐bis(2,3,4,5,6‐pentafluorophenyl)‐nickel(II) porphyrin **58** (Figure 19a).^[155]^ In the absence of an axial ligand, the complex **58** is at the antimagnetic LS state (*S* = 0) with an empty *d*_*x*_^*2*^_*‐y*_^*2*^ and a *d*_*z*_^*2*^ orbital of the central nickel ion. Upon axial ligation with at least one pyridine derivative, electrons are transferred from the *d*_*z*_^*2*^ orbital to the *d*_*x*_^*2*^_*‐y*_^*2*^ orbital, producing a paramagnetic high‐spin material (*S* = 1). The change of the spin state between *S* = 0 and *S* = 1 is achieved by complexation or de‐complexation of the pyridine derivative molecule with the square planar nickel (II) porphyrin (Figure 19b). In this process, the strong electric field between the nanoelectrodes plays a key role in the coordination reaction, and the geometrical change of the porphyrin ring and the switch of the spin state of the Ni(II) ion cause the change of the conductance.

In addition to the above design strategies, Chevallier et al. recently realized room temperature spin state switching using a reversible folding motion of Ni(II)‐porphyrin linked to an imidazole ligand via a flexible electron‐responsive mechanical hinge.^[156]^ In this system, the ligand of the 4,4′‐bipyridine unit undergoes a large amplitude and fully reversible folding motion in response to electrical stimulation, while the redox reaction of the Ni (II)‐porphyrin complex **59** can be used to remotely control the attachment of the nickel center (Figure 19c). A dimerization reaction between electrically generated bipyridyl cation radicals drives the axial coordination of additional imidazole ligands to the square planar Ni (II) center, resulting in a shift of its spin state from LS (*S* = 0) to HS (*S* = 1) with a switching efficiency of 80% (Figure 19d). The reversibility of the redox‐triggered folding motion of complex **59** was checked by thin‐layer spectro electrochemistry, as shown in Figure 19e, after 10 consecutive reduction (*E*_app_ = −0.85 V) and reverse oxidation (*E*_app_ = 0 V) cycles, no fatigue of the system was observed, and the absorption at 430 and 446 nm was fully recovered throughout the process.

## 6 MECHANICALLY RESPONSIVE SYSTEMS

Mechanical stimulation is also a common and effective form of stimulation in nature. A growing number of studies have shown that using mechanical stimulation to achieve structural changes in the molecular assembly of organic or metal‐organic complexes is a powerful and versatile tool for the design of functional materials.^[157]^ Usually, the main forms of mechanical force are mechanical grinding, crushing, rubbing, extrusion, stretching, shearing, compression, tension, and so forth, resulting in changes in the internal structure or external shape of these organometallic complexes or molecular crystals.[^1^, ^157^] The M‐L bonds are easily broken under the stimulation of mechanical forces, and the broken bonds can be recombined under certain conditions after the removal of the mechanical forces. These processes typically result in changes in the photophysical properties of organic and organometallic fluorophores, including solubility, melting temperature, external shape, elasticity, color, luminescence, quantum yield, and emission lifetime.[^157^, ^158^] Therefore, coordination complex materials with mechanically stimulated response (e.g., mechanically color‐changing materials, mechanically stimulated luminescent materials, mechanically responsive crystals, etc.) would be ideal candidates for sensors, memories, information displays, and security functions.[^3^, ^159^]

### 6.1 Mechanochromic bistable coordination complexes

**FIGURE 20:**
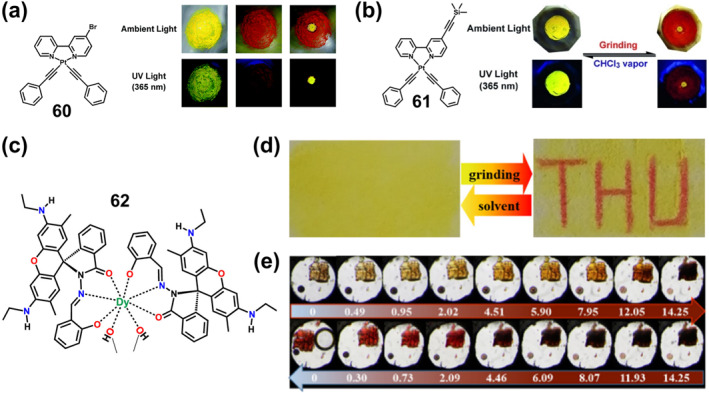
(a) Chemical structure of complex **60** (left) and its photographic images of **60‐2(CH2Cl2)** in response to mechanical grinding under ambient light and UV light irradiation (365 nm) and its reverse process (right). Reproduced with permission: copyright 2014, Royal Society of Chemistry.^[163]^ (b) Chemical structure of **61** (left) and its reversible color and luminescence changes triggered by mechanical grinding and heating (right). Reproduced with permission: copyright 2014, John Wiley and Sons.^[164]^ (c) Chemical structure of **62**, (d) the reversible color change upon grinding and solvent fuming, (e) Micrographs of **62** under different pressures. Reproduced with permission: copyright 2021, American Chemical Society.^[165]^

Mechanical color change is a reversible color‐changing switch triggered by a mechanical stimulus, which can change the color of a compound upon mechanical milling and restores to the original state by another signal perturbation.^[161]^ The mechanism of mechanical color change relies on molecular arrangement, flexibility in conformation, reversible isomerization processes, and intermolecular interactions such as hydrogen bonding, metal‐metal contacts, and aromatic π‐π stacking.^[162]^ Mechanochromic metal complexes have received significant attention due to their wide range of applications in optical recording, storage, sensing, and display devices. For example, Ni et al. reported a unique square‐planar diimine‐platinum(II) complex **60** (Figure 20a) with thermochromic and mechanochromic properties.^[163]^ They first obtained two yellow‐green crystals **60‐2(CH**_**2**_ **Cl**_**2**_ **)** and **60‐2(CHCl**_**3**_ **)** in the corresponding dichloromethane and chloroform solutions. When crystals **60‐2(CH**_**2**_ **Cl**_**2**_ **)** or **60‐2(CHCl**_**3**_ **)** were heated or ground, the bright yellow‐green emission originally centered at 525 (549, sh) nm changed to 637 and 690 nm, which corresponded to response displacements of about 88–112 nm and 141–165 nm for thermochromism and mechanical discoloration, respectively. As shown in Figure 20a, mechanical grinding triggered the emission of **60‐2(CH**_**2**_ **Cl**_**2**_ **)** reversible color and luminescence changes under ambient and UV (365 nm) irradiation, switching from yellow‐green to red after grinding, and then from red to yellow‐green after the addition of a drop of CH_2_Cl_2_ to the ground sample.

On this basis, they further synthesized a new mechanically ground or heated dual‐stimulus‐responsive luminescent diimine platinum(II) complex **61** [Pt(BpyC≡CSiMe3)(C≡CC6H5)2] (Figure 20b).^[164]^ The resulting crystals **61‐2(CHCl**_**3**_ **)** exhibit synchronous, naked eye‐perceptible and reversible color and luminescence changes. When ground or heated, its color and luminescence changed from yellow to red. At the same time, the red sample reversibly changed back to **61‐2(CHCl**_**3**_ **)** due to the absorption of CHCl_3_ vapor (Figure 20b). Through theoretical calculations, it is proved that this thermochromic and mechanochromic discoloration originates from the conversion between a triplet MLCT/ligand‐to‐ligand CT (^3^MLCT/^3^LLCT) emission state and a triplet metal–metal‐to‐ligand CT (^3^MMLCT) state.

In addition to the mechanochromic fluorescence response triggered by change in intermolecular interactions or MLCT, the use of structural transformations of ligands under mechanical forces is also an effective strategy. For instance, Kou and co‐workers reported the Dy (III) complex **62** (Figure 20c) containing the rhodamine 6G salicylaldehyde hydrazone ligand.^[165]^ With a slight grinding only, the color of the complex changed from yellow to red, which was due to the isomerization of the spirolactam C‐N bond of the rhodamine 6G part from a distorted ring‐closed structure to a planar ring‐open structure (anthracene) under mechanical stimulation. Such a transformation is fully reversible and the color of the complexes is restored after a methanolic atmosphere treatment (Figure 20d). The authors further verified the hydrostatically triggered color change, where the color of the sample changed from yellow to orange as the pressure increased from 0 to 11 GPa due to the increased intermolecular interactions at high pressure. During the decompression process, the crystal can gradually change back to its original yellow color (Figure 20e). These properties indicate the potential application of complex **62** in the field of inkless writing and force sensors.

### 6.2 Mechanoresponsive luminescent bistable coordination complexes

Mechanically responsive luminescence materials exhibit significant changes in photophysical properties, including luminescence color, intensity, or lifetime, caused by mechanical stimuli.^[160a]^ With the rapid development of the field of solid‐state photoluminescence, including fluorescence, room temperature phosphorescence, thermally activated delayed fluorescence (TADF), white light emission, and CPL, a number of soft crystals exhibiting Mechanochromic luminescence (MCL) behaviors have been reported in the past decade.^[166]^ MCL behaviors refers to mechanical stimuli with reversible changes in luminescence color, where such recovery conditions are often achieved by other external stimuli, for example, temperature treatment or exposure to solvent vapor.[^161^, ^166^] A variety of metal complex systems such as Zn(II),^[168]^ Au(I),^[169]^ Pt(II),^[170]^ Ag(I),[^109^, ^170^] Cu(I),^[172]^ and Ir(III)^[173]^ complexes) have been reported to exhibit excellent MCL responsive properties, which show strong attraction for applications such as information recording, force sensing, anti‐counterfeiting applications. However, most of these materials only change their fluorescence color under mechanical stimulation, and do not directly achieve fluorescence “on” and “off”. Only a few pure organic compounds have this property, and they are usually compounds with AIE properties. Moreover, the mechanical stimulation method is limited, mainly focusing on manual grinding.

**FIGURE 21:**
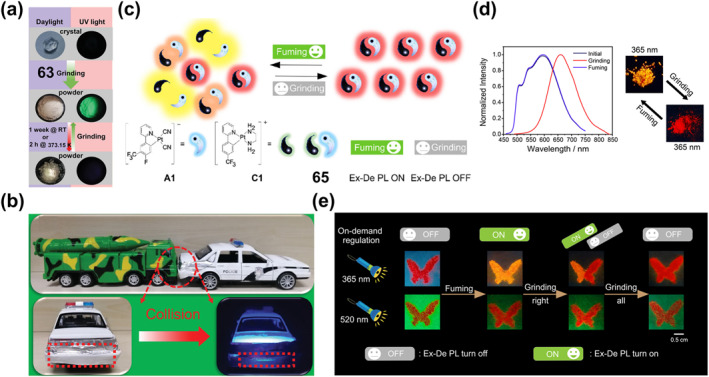
(a) The change in color or luminescence of compound **63** after grinding. (b) Car wrapped by the composite film under UV light after a collision scene; Reproduced with permission: copyright 2021, John Wiley and Sons.^[174]^ (c) The structure of **65**. (d) Normalized photoluminescent (PL) spectra of **65** in the initial, grinding, and fuming states and their accordingly luminescence photos. (e) Luminescence photos of reversible on‐off switching of the Ex‐De emission of the printed pattern using **65** solids. Reproduced with permission: copyright 2021, American Chemical Society.^[175]^

To address this issue, Fan et al. reported two hybrid metal halides, (Bmpip)_9_Pb_3_Zn_2_Br_19_(**63**) and (Bmpip)_9_Pb_3_Cd_2_Br_19_(**64**), which achieve reversible on and off fluorescence in response to both grinding and hydrostatic pressure.^[174]^ Mechanical grinding experiments showed that the crystals of **63** and **64** did not fluoresce under 370 nm excitation, while after grinding, their powders both exhibited bright green luminescence accompanied by distinct emission peaks at maximum wavelengths of 530 and 545 nm. They have excellent stability; for instance, the PL of **63** quenched after 1 week at room temperature or 2 h at 373.15 K) (Figure 21a). Also, they exhibited good solvent resistance (neither water nor organic solvent quenched the PL) and reversibility (no decrease in PL integral intensity of both compounds after 10 cycles). Hydrostatic pressure experiments showed that the PL intensity increased monotonically with increasing pressure load. The authors prepared composite films by combining this ligand material with polydimethylsiloxane for wrapping the body, which can be applied to record stress and mechanical damage after a car accident (Figure 21b).

While great progress has been made in single fluorescent color changes or on/off type switches in response to mechanical stimuli, the development of on‐demand multicolor displays is of particular importance in a variety of photonic fields, especially in optical anti‐counterfeiting applications. To achieve this goal, Li et al. developed novel material systems with controllable excitation wavelength‐dependent (Ex‐De) emission behavior by constructing Pt(II)‐based soft salts based on metal‐metal interactions and controlling the Pt(II)‐Pt(II) distance by mechanical force.^[175]^ They prepared Pt(II) complex‐based phosphorescent soft salts **65** (Figure 21c), where the distance between the two ion‐pair complexes can be easily controlled by external mechanical stress and features Ex‐De properties. The maximum emission peak of **65** in the PL spectrum was red‐shifted from 595 to 644 nm under different wavelength excitation of the initial **65** powder in the range of 360–520 nm, accompanied by a clear shift of the emission color from yellow‐orange to red. After grinding, due to the strong Pt(II)‐Pt(II) and π‐π interactions between the two oppositely charged complexes, the PL band of **65** powder under 365 nm excitation was red‐shifted from bright orange to dark red (Figure 21d). However, after grinding, the Pt(II)‐Pt(II) distance of **65** becomes increasingly similar due to the isotropic pressure, and the emission peak of **65** powder remains almost unchanged when excited at 520 nm, suggesting that its Ex‐De emission behavior has been deactivated. After acetone vapor treatment, the emission color of **82** powder can be restored (from red to orange‐yellow), indicating that the Ex‐De emission behavior is activated (Figure 21e). By controlling the Pt(II)‐Pt(II) distance of **65** through mechanical stress and vapor fumigation, the authors achieved the first reversible switch with Ex‐De emitting properties and demonstrated its advanced anti‐counterfeiting application with an on‐demand multicolor display.

**FIGURE 22:**
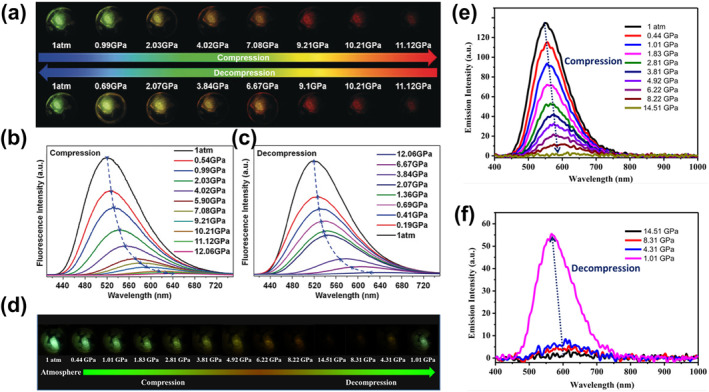
(a) Photographs of **66** during a compression–decompression cycle under UV illumination at 365 nm. In situ emission spectra upon (b) compression and (c) decompression. Reproduced with permission: copyright 2019, John Wiley and Sons.^[176]^ (d) The fluorescence image of **69** at different pressures; Fluorescence emission spectra of powder **69** during (e) compression and (f) decompression in a diamond anvil cell. Reproduced with permission: copyright 2022, American Chemical Society.^[177]^

Although multicolor displays under mechanical stimuli can be achieved by introducing fluorophores with Ex‐De properties, these smart luminescent materials have some limitations. For example, most of them exhibit abrupt two‐color switching instead of smooth wavelength shifting, and a second force is always required to reverse the change, which is difficult to control. To address this, Bu's group designed and reported a continuous, reversible, mechanical response‐driven coordination network that correlates linearly with proportion to the stimulus.^[176]^ They obtained coordination networks **66** with gradual reversible structural deformation under both thermal and pressure stimulation by coordinatively binding tris(4‐(pyridine‐4‐yl)phenyl)amine (TPPA) molecules to the channels in a face‐to‐face manner. The emission color of the crystals of **66** gradually changed from green (525 nm) to orange (585 nm) to red (620 nm) as the pressure changes from 1 atm to 11.12 GPa (Figure 22a), and the emission intensity monotonically decreased and the fluorescence quenched when the pressure reaches above 11.12 GPa. The emission maxima and intensities of the whole process exhibit linear dependence on the pressure. Moreover, the fluorescence changes of **66** under pressure stimulation have excellent reversibility (its fluorescence turns on again and recovers after decompression) and good cyclability (fluorescence remains reversible for 10 consecutive compression‐decompression cycles) (Figures 22b,c). The increasing pressure leads to a sharp contraction of the lattice of **66**, with the central N atom re‐hybridizing from sp^3^ to sp^2^ and tending to planarize, allowing closer proximity between TPPA molecules, reducing the spatial site resistance and greatly enhancing π‐π interactions, and decreasing fluorescence emission. This rare multiple stimuli‐response luminescence tuning behavior arises from the change in conformation and alignment under mechanical stimulation, achieving a linear function of stimulus intensity with reversible fluorescence changes and a wide range of wavelength shifts, which provides new insights into stimuli‐responsive luminescent materials.

Aggregation‐induced emission complexes are also an important alternative source for mechanical chromic luminescence complexes.^[178]^ M‐L interactions can drive the spontaneous formation of M‐L bonds between organic donors and metal acceptors.^[179]^ Therefore, this coordination‐driven self‐assembly strategy is expected to be useful for constructing supramolecular coordination complexes (SCCs) with AIE properties.^[180]^ In a recent example, Yin et al. synthesized supramolecular platinum(II) metallocycles with AIE enhancement (AIEE) properties by ligand‐driven self‐assembly of bis‐platinum(II) acceptors and organic donors, which exhibit high‐contrast mechanochromic fluorescence.^[177]^ They synthesized three supramolecular hexagonal fluorescent platinum(II) metallocycles **67–69** modified by TPA, carbazole, and tetraphenylethene, respectively, through a directed bonding strategy for constructing SCCs with bright aggregation states and mechanostimuli‐responsive luminescence. There are some differences in the properties of these complexes, with complex **67** exhibiting AIEE but no mechanofluorescence, whereas carbazole‐ or tetraphenylethylene‐modified complexes **68** and **69** exhibit bright fluorescence both in dilute CH_2_Cl_2_ solutions and in various aggregation states. Moreover, the complexes **68–69** also exhibited unique mechanofluorescence properties. Specifically, compound **68** displayed weak mechanochromic fluorescence, while compound **69** exhibited high‐contrast reversible mechanofluorescence (Figures 22d–f). Such rare metallocycles with AIEE, aggregated fluorescence change, or mechanochromic fluorescence properties have potential applications in cell imaging and solid‐state illumination.

### 6.3 Mechanically triggered magnetic switch based on bistable coordination complexes

Some complexes containing transition metal ions exhibit switchable magnetic double‐stable spin‐crossover (SCO) phenomena, which are driven mainly by the rearrangement of coordination spheres of metal ions in response to external stimuli.^[181]^ After persistent efforts by scientists, selective control of SCO of individual molecules has been achieved.^[182]^ For example, developing mechanical control of single‐molecule SCO by mechanically controlled break junctions and scanning tunneling microscopy (STMs) techniques.^[182a]^ Lin and coworkers developed a method to mechanically induce reversible SCO of single Fe‐porphyrin (FeP) molecules via the scanning tunneling microscope break junction technique.^[181a]^ In the FeP single‐molecule junction formed under STM, the zero‐bias resonance associated with the Fe center changes its line shape as the gap width changes. Thus, the Fe spin state of a single FeP molecule can be changed by adjusting the tip height by mechanical stretching or squeezing the joint. When the junction gap is narrow, the macrocyclic nucleus of the porphyrin exhibits a saddle structure with a HS (*S* = 2) Fe center. As the junction gap widens, the molecule is stretched and the macrocyclic nucleus gradually flattens, changing from a saddle to a planar conformation where the 3% Fe‐N bond is shortened, leading to the formation of an intermediate spin (*S* = 1) Fe center. This process is fully reversible and can be repeated many times.

### 6.4 Mechanical induced reversible single crystal to single crystal phase transitions in bistable coordination complexes

**FIGURE 23:**
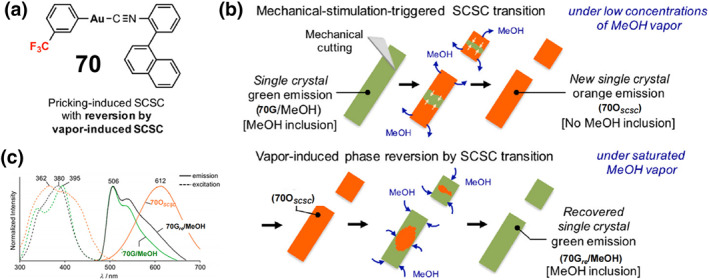
(a) Molecular structure of complexes **70**. (b) Mechanical‐stimulation‐triggered single crystal to single crystal (SCSC) transition (up) and its reversed SCSC transition upon exposure to a saturated MeOH vapor environment (bottom). (c) Emission spectra (λex = 365 nm) and excitation spectra of **70‐G/MeOH** (green solid line), **70‐Gre/MeOH** (black solid line), and **70‐Oscsc** (orange solid line). Reproduced with permission: copyright 2018, American Chemical Society.^[185]^

Single crystal to single crystal transformations triggered by mechanical stimuli are an interesting phenomenon in molecular crystals, and although SCSC transitions are usually difficult, they can provide detailed information about changes in structural and/or emission properties at the molecular level.[^159^, ^182^] For example, Seki and Ito et al. reported the first example of mechanically induced single‐crystal‐to‐single‐crystal (SCSC) phase transitions of Au(I) isocyanide complexes accompanied by changes in emission color.[^182^, ^183^] However, these mechanically stimulated triggered SCSC phase transitions can only drive the transformation of sub‐stable polycrystals into thermodynamically more stable polycrystals, and the reverse phase transition of crystals after mechanical stimulation has not yet been realized. For this reason, after continuing efforts, in 2018, their group reported the first luminescent complex with a reversible mechanical stimulus‐triggered SCSC transformation, that is, a green crystalline gold(I) isocyanide complex **70** (Figure 23a) bears CF_3_ and biaryl moieties.^[185]^ The crystals of gold(I) complex **70** were cut under methanol (MeOH) vapor at 295.15 K, where MeOH was simultaneously released and the green luminescent single crystal was spontaneously transformed into a centrosymmetric orange luminescent single crystal (P1). After treatment under saturated MeOH vapor, the complexes can reversibly recover from orange‐emitting crystals to green‐emitting (Figures 23b,c). This design strategy depends mainly on the SCSC transition triggered by mechanical forces and solvents between polar crystals containing a polar solvent (MeOH) and centrosymmetric crystals without solvent molecules.

## 7 OTHERS

In addition to the above shown stimuli responsive smart coordination systems triggered by external energy, that is, light, temperature, electricity, and mechanical force. There are also non‐energetic stimuli such as chemical stimuli like pH, solvents, and additive chemicals. With delicate design, some coordination complexes can undergo structural transformation or coordination form alteration under these chemical stimuli, providing an effective pathway for smart‐responsive systems. Notably, some smart bistable coordination complexes are multi‐addressable and can respond to multiple stimuli and produce multiple states under different stimuli, which broadens the application areas of smart materials. Therefore, in this section, we will focus on pH‐responsive systems, solvent‐stimulus‐responsive systems, and multi‐addressable coordination systems.

### 7.1 pH responsive systems

As a key chemical stimulus, pH plays a crucial role in many chemical and biological processes. Most M‐L complexes are sensitive to pH, and thus the resulting pH‐responsive coordination systems have promising applications in biology and biomedicine, sensors, intelligent robotics, etc.^[186]^ pH‐responsive metal composites usually change its own morphology or physicochemical properties in response to changes in the external pH, and the most classical example is acidochromic behavior.^[187]^

**FIGURE 24:**
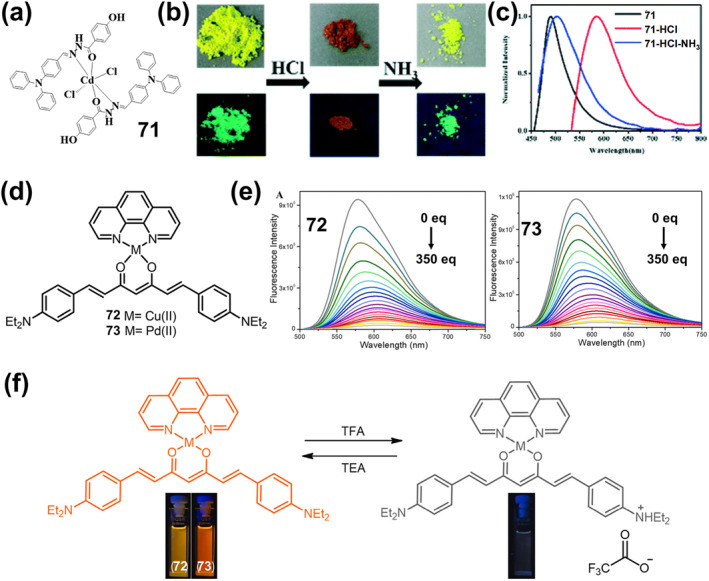
(a) The structure of complexes **71**; (b) Images of complex **71** in different solid states under ambient light and the irradiation of UV light at 365 nm. (c) Emission spectra of complex **71** in different solid states; Reproduced with permission: copyright 2022, Royal Society of Chemistry.^[190]^ (d) Metal complexes **72** and **73**, (e) Absorption spectra of complexes (**72** and **73**) in CHCl_3_ upon gradual addition of trifluoroacetic acid (TFA) (0–350 eq). (f) Photograph of the corresponding solution in CHCl3 under UV irradiation 365 nm before (fluorescence on)/after (fluorescence off) addition of TFA (350 eq) and reversible fluorescence upon addition of TEA. Reproduced with permission: copyright 2022, Elsevier.^[188c]^

These acidochromic complexes undergo significant changes in absorption or emission in response to acid‐base stimuli.^[188]^ The major factor in the acidochromic properties of the complexes include protonation and deprotonation processes, interruption of solvent molecule‐ligand interactions, and disintegration and reaction with the complexes due to acid adsorbed on the surface of the crystals.[^167^, ^187^] The representative structures are metal complexes based on Schiff base ligands, which have received much attention because of their simple synthesis, easy structural modification, and strong chelating ability for metal ions.[^167^, ^187^, ^188^] For example, Zheng's group synthesized an acetonitrile solvate of Schiff base molecule (HL) with acetonitrile (HL‐2CH_3_CN) and its Cd(II) complex (Cd(HL)_2_Cl_2_, **71**) (Figure 24a) and realized mechanochromic and acid‐base chromogenic systems with high contrast.^[190]^ Owing to both coordination bonds and molecular HL‐rich hydrogen bond donors and acceptors, the Cd(II) complex has different emission peaks and luminescence properties from HL‐2CH_3_CN. On the one hand, the phase transition from the crystalline to the amorphous state allows the complex **71** to exhibit a high‐contrast mechano‐chromatic luminescence. More importantly, complex **71** has significant acid‐base responsive properties. After treatment with HCl vapor (36% concentrated hydrochloric acid vapor), the color of the sample changed from yellow‐green to orange, while the fluorescence emission color changed from cyan to orange, named as **71‐HCl**. When **71‐HCl** was further fumigated with NH_3_ vapor (25% concentrated ammonium hydroxide vapor), the color and emission color of the sample (named **71‐HCl‐NH**_**3**_) were restored (Figures 24b,c). The authors demonstrated by XPS that the acidochromic properties of the complexes are attributed to the protonation of the ‐NH‐ groups. After treatment with NH_3_ vapor, the samples undergo deprotonation, resulting in the recovery of fluorescence. However, the HL‐HCl sample cannot be restored to its initial state due to the serious collapse of the original crystal structure and the departure of acetonitrile molecules in the protonation and deprotonation process, thus preventing reversible transformation under acid‐base stimulation.

For biomedical applications, acidochromic fluorescent properties have become more attractive, such as cell imaging with fluorescent properties, and cellular damage from exposure to visible light. In particular, curcumin and its derivatives are of interest due to their environmental sensitivity and optical properties. However, its free molecules have low photoactivity, which limits their use in photodynamic therapy. To overcome this limitation, de França et al. proposed a strategy for chelation of D‐π‐A‐π‐D curcumin dyes with the metals Cu (II) and Pd (II) and prepared two highly photoactive metal complexes, **72** and **73** (Figure 24d), by combining them with the bidentate ligand 1,10‐phenanthroline.^[188c]^ The metal complexes (**72** and **73)** showed significantly higher single‐linear oxygen quantum yields (*Φ* = 0.36 and 0.54) compared to the curcumin ligands and had distinct acid‐stimulated fluorescence transition properties. When no trifluoroacetic acid (TFA) was added, the complexes **72** and **73** show orange fluorescence (578 nm). After the gradual addition of TFA, the fluorescence intensity of the metal complexes slowly decreased with a red‐shifted emission band (610 nm) accompanied by a change in emission color to colorless (Figure 24e). Interestingly, this process can be completely reversible upon the addition of TEA for neutralization (Figure 24f).

### 7.2 Water‐triggered responsive systems

Water is a common resource that is low cost, easy to use, green and clean, and is one of the most attractive ways to create a stimulus. Particularly for materials designed for environmental protection, water provides clean, energy‐saving and reversible benefits. For example, hydrochromic materials and hydrochromic fluorescence materials can change color or fluorescence under the stimulation of pure water^[191]^ or moisture,^[192]^ and restore the initial state by heating stimulation or adding other compounds, and are widely used in recyclable water‐jet rewritable printing^[193]^ and anti‐counterfeiting materials.^[194]^ Organic‐inorganic metal halide hybrids have been used as highly promising materials for rewritable paper due to ease of processing, structural tunability, and inherent mechanical flexibility. However, the potential structural transformation mechanism of small molecule‐induced PL quenching has rarely been investigated in these materials, which limits their extended application in rewritable paper. On the other hand, current rewritable papers still have problems and limitations such as tedious and time‐consuming preparation, requirement of an initial photomask of photochromic materials, and cumbersome printing methods. It is a challenge to simplify the structure of rewritable paper to realize repeated printing and erasing under mild conditions.

**FIGURE 25:**
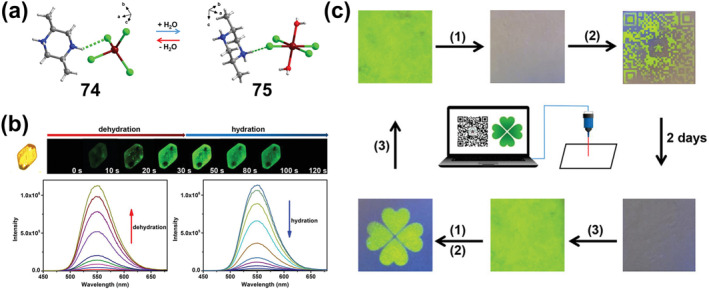
(a) The single crystal to single crystal (SCSC) transformation between **74** and **75** in the hydrated and dehydrated process; (b) Photographs of crystals (**74**, **75**) under UV irradiation (up) and the emission spectra of complex **75** measured sequentially in hot stage at 333.15 K and water vapor filled atmosphere (RH ≈ 43% at 298.15 K) (c) Photographs of the rewritable photoluminescent (PL) paper under UV irradiation. Procedures: 1) exposing to moisture environment for erasing; 2) laser printing imaging; 3) annealing at 393.15 K for initialization; Reproduced with permission: copyright 2021, John Wiley and Sons.^[195]^

To solve these problems, Zang's group realized PL rewritable papers in response to water molecule stimulation by introducing organomanganese bromide hybrids with excellent PL centers.^[195]^ They first prepared two 0D organobrominated manganese hybrids, C_6_N_2_H_16_MnBr_4_(**74**) and C_6_N_2_H_16_MnBr_4_(H_2_O)_2_(**75**), using *trans*‐2,5‐dimethylpiperazine as the organic cation. The organometallic halide hybrid **74** exhibits green luminescence with a PL quantum efficiency of 82%. The complexes were non‐emissive when transformed into the hydrated phase **75** by absorption of a ligand water molecule, in which Mn^2+^ adopts a quasi‐octahedral coordination sphere (Figure 25a). This reversible transition between the hydrated and dehydrated phases led to a reversible single‐crystal to single‐crystal (SCSC) structural transition producing an on‐off fluorescence (green emission **74** and non‐emission **75**) (Figure 25b). The authors further coated the filter paper with compound **75** to obtain rewritable PL paper, realizing an environmentally friendly rewritable paper with excellent “write‐erase‐write” recycling capability (Figure 25c). This research on PL performance changes through phase transitions provides a new approach to water‐responsive metal halide hybrids for rewritable paper.

**FIGURE 26:**
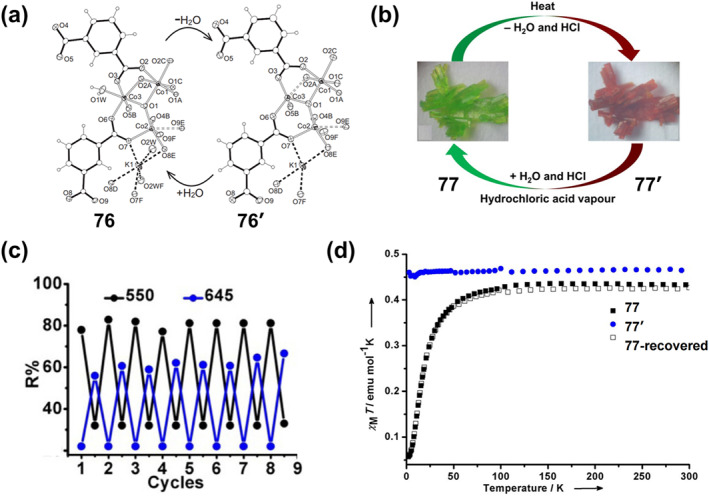
(a) Coordination environments of **76** and **76′**. Reproduced with permission: copyright 2007, John Wiley and Sons.^[196]^ (b) Reversible single crystal to single crystal (SCSC) transformation between **77** and **77′**. (c) The desorption/absorption curves of complex **77** in H_2_O and HCl for 8 cycles were measured by recording the peak changes at 550 and 645 nm. (d) *χ*_M_ *T* versus *T* plots under a 1 kOe field for **77** and **77′** and the recovered sample. Reproduced with permission: copyright 2017, American Chemical Society.^[197]^

In addition to PL changes induced by water‐stim ulated SCSC dynamic transitions, some porous magnets with guest‐dependent spin‐crossover properties are accompanied by reversible magnetic behavior. A classic example is aflexible three‐dimensional porous magnet [KCo_7_(OH)_3_(ip)_6_(H_2_O)_4_]‐12H_2_O (**76**) (H2ip is isophthalic acid) reported by Chen.^[196]^ When a water molecule acts as a guest, this complex can undergo a reversible SCSC transition in response to adsorption and desorption of water molecules and has magnetic behavior modulation properties (Figure 26a). This single crystal **76** was heated to 393.15 K to obtain dehydrated [KCo_7_(OH)_3_(ip)_6_](**76′**) single crystals accompanied by a color change from red to purple. When the dehydrated purple crystals were exposed to air for a few days, the crystal color returned to red. At low temperatures, the dc susceptibility (χ_M_ *T*) value of **76′**, although also increasing with the applied magnetic field, showed a much weaker magnetic response than that of **76** at the same magnetic field due to competition effects. The absence of peaks in the AC magnetization of **76′** confirms that the superparamagnetic behavior of **76** is at least partially contributed by disordered solvent molecules. By exposing **76′** to air for several days to obtain rehydrated samples, it was found that the magnetic characteristics of **76** were fully restored in the rehydrated samples, suggesting that the magnetic properties of **76** and **76′** can be reversibly regulated by guest desorption/adsorption. This transformation is mainly due to a change in coordination geometry due to the removal of solvent molecules, but there is no significant lattice change. Such complexes exhibiting reversible single‐crystal‐to‐single‐crystal transition characteristics can alter their magnetic properties through gas‐phase adsorption/desorption, finding extensive applications in areas like switches or sensors. Nevertheless, achieving SCSC transformation through vapor adsorption/desorption in nonporous solids is highly challenging as this process typically results in the formation of crystalline powders in the solid state.^[198]^

To address the aforementioned issues, Wang and colleagues innovatively designed nonporous Cu(II) complexes, leveraging the benefits of nonporous materials to reconcile the inherent conflict between magnetic exchange and porosity.^[197]^ The team synthesized a novel discrete nonporous copper(II) complex **77** {(H_3_O)[K(15‐crown‐5)_2_][Cu^II^Cl_4_]} via the reaction of CuCl_2_, 15‐crown‐5, and KCl in water in a 1:1.5:1 M ratio. They further determined by single‐crystal X‐ray diffraction structural analysis that compound **77** crystallizes in the triclinic *P*ī space group with an asymmetric unit containing two isolated [CuCl_4_]^2‐^ anions, two [K(15‐crown‐5)_2_]^+^ cations and two hydrated protons. Also, complex **77** can undergo a single‐crystal‐to‐single‐crystal (SCSC) transformation to yield another discrete nonporous compound **77′** {[K(15‐crown‐5)2][Cu^II^Cl_3_]}.This transformation occurred through thermally‐induced desorption of H_2_O and HCl, followed by recovery through absorption of H_2_O and HCl (Figure 26b). Through monitoring diffuse reflectance UV‐vis data at 550 and 645 nm, they observed that the transition between **77** and **77′** could be effortlessly repeated for a minimum of 8 cycles during the desorption/absorption of HCl and H_2_O (Figure 26c). The SCSC transition is accompanied by notable alterations in coordination number (4 ↔ 3), space group (*P*ī ↔ *P*2_1_/*c*)), and color (green ↔ red). Moreover, this phenomenon is not undergoing a crystalline‐powder or single‐crystal‐powder transition during the chemisorption/desorption of gaseous HCl, and instead, it is due to the fact that compounds **77** and **77′** constitute a new family of nonporous molecular crystals displaying the chemisorption/desorption of gaseous HCl. Owing to the substantial alteration in the coordination geometry of the Cu(II) center, the magnetic properties (antiferromagnetic ↔ paramagnetic) exhibit reversible changes, as evidenced by the examination of temperature‐dependent magnetization data in **77** and **77′**, along with the change in *χ*_M_ *T* of the recovered samples (Figure 26d). Due to the presence of weakly antiferromagnetic Cu‐‐‐Cu interactions through the Cu‐Cl‐‐‐H_3_O‐‐‐Cl‐Cu hydrogen bonds, compound **77** shows a *χ*_M_ *T* value of 0.44 emu K mol^−1^ at 300 K, and a sharp decrease to 0.055 emu K mol^−1^ at 2 K (Figure 26d). While **77′**, the *χ*_M_ *T* value is almost constant at 0.48 emu K mol^−1^ over the entire temperature range, behaving paramagnetically. This is due to the fact that after the release of H_2_O and HCl, the Cu^II^ ion has a long distance (9.0971(5) Å) from the nearest Cu‐‐‐Cu without any bridging. This complex holds promising prospects for applications in the development of devices like chemical switches or sensors.

### 7.3 Organic solvent stimuli‐responsive systems

**FIGURE 27:**
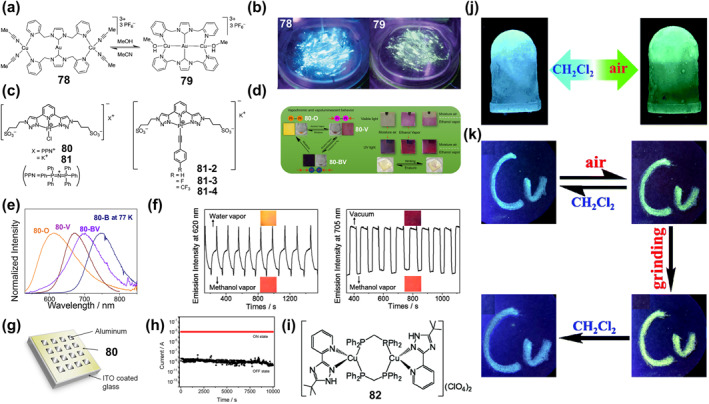
(a) Chemical structure of complexes **78** and **79**; (b) photographs of complexes **78** and **79** by treating with MeOH(g) or MeCN(g); Reproduced with permission: copyright 2010, American Chemical Society.^[200]^ (c) Structures of the complexes **80**, **81** and its derivers. (d) Photographic images of the reversible color changes associated with the vapochromic phenomenon, vapoluminescent phenomenon, and fast writing and erasure application in complexes **80** in solid and thin film states. (e) Normalized emission spectra of complex **80** solid in different forms at room temperature. (f) Responsive times and cycle numbers for reversible vapoluminescent property of complex **80** when exposed to methanol and water vapor(left) and methanol vapor and vacuum(right) alternately. (g) Schematic diagram of the device structure. (h) Retention time of the memory device at ON and OFF states under a constant voltage stress at 1 V. Reproduced with permission: copyright 2017, American Chemical Society.^[201]^ (i) Chemical structure of complex **82**. (j) Luminescence images of **82**‐coated LED chip under CH_2_Cl_2_ vapor and in air. (k) Fluorescence variation of the word “Cu” written in **82** under CH_2_Cl_2_ vapor, in air, after grinding, and re‐exposure to CH_2_Cl_2_ **vapor**. Reproduced with permission: copyright 2022, Royal Society of Chemistry.^[202]^

Some transition metal complexes could form solvent‐sensitive products through the association and dissociation of metal‐solvent bonds, and often undergo reversible property shifts in response to organic solvents (especially volatile solvent vapors), such as color and/or luminescent color.^[199]^ A classic example of vapor chromogenesis is an interesting Au(I)‐Cu(I) complex system, Au{im(CH_2_py)_2_}_2_{Cu(MeCN)_2_}_2_][PF_6_]_3_(**78**) (Figure 27a), reported by Catalano and Strasser.^[200]^ This ternary compound **78** has blue emission and gives access to the green‐emitting [Au(im(CH_2_py)_2_)_2_(Cu(MeOH))_2_](PF_6_)_3_ (**79**) after liquid MeOH or MeOH vapor treatment. This is because the substitution of the methyl cyanide (MeCN)molecule coordinated to the Cu(I) center by the MeOH molecule causes a change in the Au(I) and Cu(I) coordination geometry leading to the formation of new Cu‐O(solvent) and Au‐Cu bonds. So this process is reversible, **79** can restore the blue emission of **78** in MeCN vapor (Figure 27b). Interestingly, **79** was able to obtain yellow‐orange (*λ*_max_ = 543 nm) emission after exposure to air due to partial loss of MeOH, while becoming yellow (*λ*_max_ = 573 nm) due to the complete loss of MeOH in vacuum, which rapidly reversed in methanol vapor. In addition to this, **78** can also produce yellow‐orange (*λ*_max_ = 591 nm) and green (*λ*_max_ = 519 nm) fluorescence upon stimulation with acetone and H_2_O, respectively. The emission change mechanism of this solvent‐chromic compound originates from the ligand substitution reaction between the solid complex and the solvent vapor, and the color change mechanism may be caused by the “on‐off” Au‐Cu interaction due to the ligand exchange reaction.

Similarly, Pt(II) complexes exhibit intriguing vapor‐chromatic properties.^[203]^ Alterations in the metal‐metal and π‐π stacking interactions of Pt(II) complexes induced by gases from VOCs result in a wealth of vapor‐chromatic and vapor‐luminescent properties. These properties are appealing for the advancement of chemical sensors.[^157^, ^203^] Nevertheless, numerous Pt(II) complexes encounter sluggish response/recovery processes and intricate procedures in vapor‐chromotropic systems. This, combined with inadequate selectivity and reproducibility, imposes constraints on their practical applications.^[205]^ Hence, it becomes crucial to devise materials with heightened sensitivity and superior selectivity toward specific substances. Additionally, these materials should demonstrate rapid response, excellent reversibility, and facile device fabrication. In tackling this challenge, Li and collaborators conceptualized and created a range of amphiphilic platinum complexes (**80**, **81**,**81‐2**,**81‐3** and **81‐4**) incorporating 2,6‐bis(1‐propanesulfonic acid‐1,2,3‐triazol‐4‐yl)pyridine (btapy) featuring a tridentate N ligand (Figure 27c).^[201]^ Notably, complex **80** demonstrates highly sensitive gas‐phase color alterations and luminescent responses to vapors of water and alcohols, including methanol, ethanol, propanol, isopropanol, and butanol. Its commendable reversibility results in profound color and luminescence alterations. An immediate and striking shift in color, transitioning from orange (**80‐O**) to violet (**80‐V**), occurred upon exposing solid samples to methanol vapors (Figure 27d). The visible absorption band experienced a red‐shift from 468 to 550 nm, while the associated emission study revealed a red‐shift from 614 to 672 nm following the adsorption of methanol vapor (Figure 27e). Even more remarkable is the discovery that removing methanol vapors from **80‐V** under vacuum or with dry nitrogen leads to an additional shift in color to blue‐violet (**80‐BV**) and a further redshift to 700 nm at the emission maxima(Figure 27e). However, **80‐BV** can transform into **80‐O** when exposed to water vapor or moisture, whereas **80‐O** cannot be converted to **80‐BV** through direct vacuum drying, even for a week or by heating. The alteration in emission intensity was observed while exposing the films to alternating methanol and water vapor or methanol and vacuum conditions. The films, formed by casting a methanol solution of **80** onto a quartz substrate, exhibited highly reversible luminescence changes with a brief response time (Figure 27f). Furthermore, this material was utilized to prepare compact solution‐processed resistive memory devices (Figure 27g), exhibiting stable binary storage capabilities, elevated on/off ratios, and extended retention times(Figure 27h). Consequently, this material, with its vapor‐chromic and vapor‐emitting properties, holds potential applications in chemical sensing, logic gates, VOC monitoring, and memory functions.

In the given examples, the solvent‐induced chromatic mechanisms manifest through alterations in Au‐Cu spacing resulting from ligand substitution reactions with solvent vapors, and adjustments in π‐π interactions and Pt‐Pt distances influenced by solvent vapors, respectively. For ion‐emitting complexes, their luminescent properties are typically influenced by electrostatic interactions between positive and negative ions. Due to the inherently weak nature of electrostatic interactions, exerting an effect on bulky ligand ions becomes challenging.^[206]^ While hydrogen bonding, being a distinct form of weak interaction, is susceptible to damage from various external forces. The disruption and subsequent reconstruction of hydrogen bonds frequently induce alterations in the packing arrangement, giving rise to a variation in luminescent color.^[207]^ Consequently, in response to this challenge, Chen's group devised a novel bicopper ion complex, orchestrating the luminescent transition by means of solvent vapor‐triggered disruption and reconstruction of hydrogen bonds.^[202]^ They documented the discovery of a freshly characterized emissive dicopper (I) complex **82** (Figure 27i), featuring bis(diphenylphosphino)methane (dppm) and 5‐*tert*‐butyl‐3‐(2′‐pyridinyl)‐1,2,4‐triazole (bptzH), showcasing a vapor‐responsive blue‐green‐yellow luminescent transition and possessing TADF attributes. Illustrated in Figure 27j, the LED chip coated with complex **82** emits blue radiation in the presence of CH_2_Cl_2_ vapor and transitions to green radiation when exposed to air, demonstrating excellent reversibility. Additionally, they employed an invisible ink composed of CH_2_Cl_2_/hexane (*v/v*, 1:10) to write the word “Cu” onto paper using suspension **82**. Following grinding, the initially green luminescent “Cu” transformed into a yellow luminescent state (Figure 27k), and exposure to CH_2_Cl_2_ vapor induced a shift to blue luminescence for “Cu” (Figure 27k). The blue‐green two‐color transition arises from the removal and subsequent reintroduction of the CH_2_Cl_2_ molecule, while the yellow‐green two‐color transition results from the disruption and restoration of the filled arrays induced by the breaking and reconstruction of NH‐O hydrogen bonds. The innovative design strategy for adjusting the color of solid‐state luminescence offers fresh perspectives for advancing the field of stimulus‐responsive smart luminescent materials.

### 7.4 Multi‐addressable molecular switch based on bistable coordination complexes

To meet the needs of applications in diverse fields ranging from molecular switches to smart materials, various multi‐addressable molecular switch architectures are being sought. Compared to single‐stimulus systems, multi‐addressable systems have more complex functions and behaviors while generating multiple states with different stimuli and possible interactions with each other.^[208]^ When multiple stimuli work in synergy, they often produce unexpected results that are not achieved with a single stimulus state.^[209]^ A feasible strategy is that photo‐responsive derivatives are combined with electronically responsive transition metal complexes, which requires that the different redox states of the metal complexes do not overlap with the electrochemical response of the photochromic unit and have fast response times.^[210]^

**FIGURE 28:**
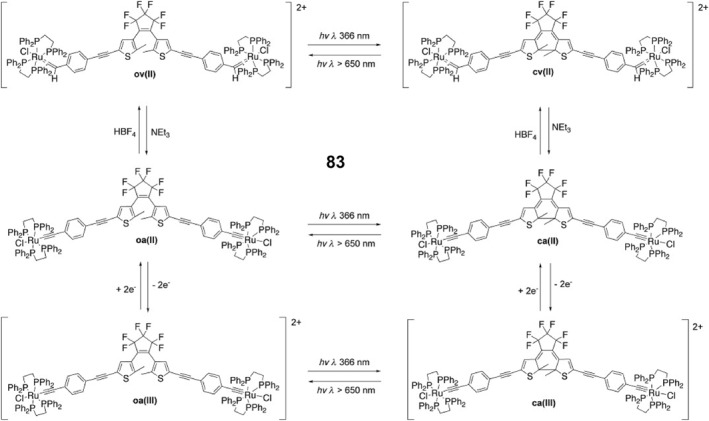
Interconversions of the different states oa(II), ov(II), oa(III), ca(II), cv(II), and ca(III) of complex **83**. o = open dithienylethene (DTE), c = closed DTE, a = alkynyl, v = vinylidene, II/III = (formal) metal‐centered oxidation states; Reproduced with permission: copyright 2009, John Wiley and Sons.^[211]^

Since molecular switches and logic‐gated bistable molecules provide states with different linear optical properties (absorbance, fluorescence) and can be interconverted by chemical, redox, magnetic, or photonic stimuli, they are ideal candidates to apply Boolean logic operations. However, the NLO properties of organometallic complex‐based molecular switches have been relatively little explored in the direction of multi‐addressing. To this end, Green et al. reported a dinuclear metal‐alkynyl complex **83** that can provide six stable and switchable states with different cubic NLO properties.^[211]^ The complex **83** consists of three independent addressable modes and is connected by an alkyne‐functionalized 5,5′‐dithienylperfluorocyclopentene (DTE) bridge. The transition of the alkyne ligand to the vinyl group exhibits proton‐responsiveness. The metal‐centered redox between Ru(II) and Ru(III) in the complex exhibits electrochemical responsiveness, and the ring opening and closing of the DTE is dependent on the photostimulus. Specifically, the complex oa(II) was reversibly protonated to the di(vinylidene) complex ov(II), then reversibly oxidized to the Ru(III) complex oa(III), and photoisomerized to the closed alkyne‐based complex ca(II) under UV light. Similarly, the complex ca(II) is reversibly protonated to cv(II), reversibly oxidized to ca(III), and photoreducted to oa(II) under red light irradiation. The vinylidene‐preferring and oxidized forms undergo reversible photoisomerization under similar irradiation conditions. Since the oxidation of the ruthenium biradical complex with these ligands is an irreversible process, the complex **83** was found to have six reversible switching states with different third order nonlinear (NLO) properties as assessed by cyclic voltammetry (Figure 28). This complex can respond to different stimuli over a wide spectral range and has great potential for constructing multi‐input logic gates.

**FIGURE 29:**
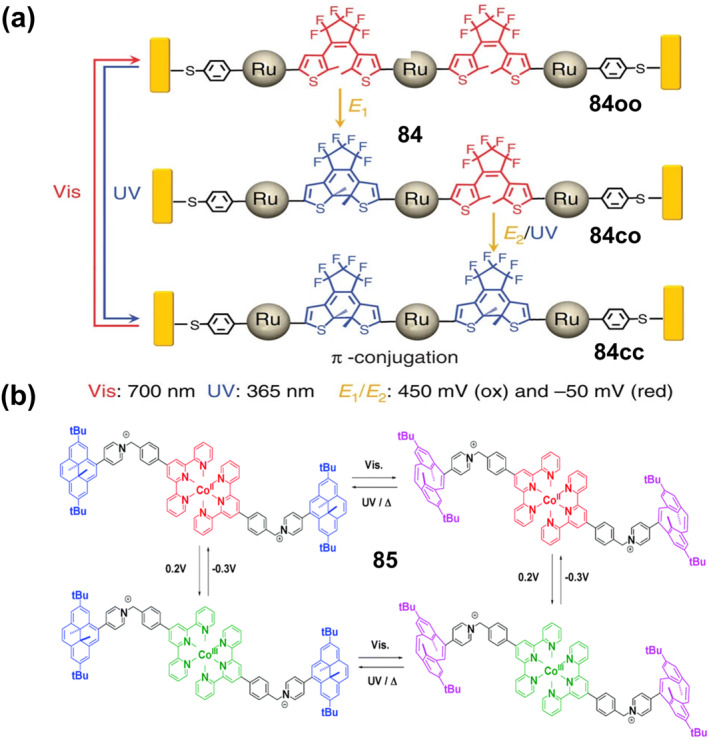
(a) Scheme of molecular isomerization of **84** under external controls. E1 and E2, two cycles of electrolysis; UV, UV irradiation; Vis, visible light irradiation. Reproduced with permission: copyright 2014, Springer Nature.^[212]^ (b) Multi‐addressable states for the complex **85** modulated using photo‐ or thermal and electrochemical redox processes. Reproduced with permission: copyright 2016, Royal Society of Chemistry.^[210b]^

In order to meet these practical applications, the development of functional materials with individual molecules as functional elements in nanodevices is of great significance, especially utilizing the synergistic effect of external stimulation and the fabrication of corresponding multi‐addressable molecular transport junctions (MTJs). Based on this, Meng et al. took advantage of the synergistic modulation of multiple external controls and reported for the first time the photo‐ and electro‐commutation of MTJs based on on‐wire lithography‐generated nanogaps modified with two Ru/diethylene (DTE) complexes to realize multi‐addressable nanodevices.^[212]^ They designed a novel multi‐addressing system based on DTE that employs two DTE units of the trimetallic complex **84** and fabricated MTJs for electrical and optical switching using nanogaps (∼5 nm) modified with this complex. Due to the very low potential of electrochemical cyclization, reversible and reproducible conductivity switching can be easily achieved by orthogonal optical and/or electrochemically triggered molecular heterodimerization. On the one hand, irradiation by visible light (700 nm) or UV light enables the isomerization of the DTE molecule between the fully π‐conjugated **84cc** with low resistance and the non‐π‐conjugated **84oo** with high resistance. On the other hand, the system also achieves a stepwise and orthogonal switching of the electrical conductivity of the nanodevice by gradually activating the two DTE units under electrochemical and/or optical stimulation (Figure 29a). Such a nanodevice based on a multi‐addressable MTJ has important applications in various logic operations.

The above examples demonstrate that the use of photochromic couples as key components of light‐energy molecular switches is one of the most attractive and straightforward strategies for developing useful molecules or materials in molecular electronics. In order to further improve the photoconversion rate, the isomerization quantum yield or the stability of the two states, attempts have been made to introduce different light‐responsive molecules. For example, with the same design strategy as above but a different photochromic molecule, Bakkar et al. reported a multi‐addressable four‐state molecular switch **85** (Co^II^(Lc)_2_^4+^) based on a dimethyldihydropyrene‐substituted terpyridine cobalt complex.^[210b]^ Among them, the ligand Lc^+^ of dimethyldihydropyrene exhibits photo‐responsive properties, enabling reversible transformation between closed structures and open isomers in response to light and/or heat, while the two different redox states Co^II/I^ and Co^III/II^ of the Co^II^ center of the complex can be reversibly switched under electrical stimulation. Since each state is stable and unambiguously identified by spectroscopic and electrochemical features, interconversion between the four stable states can be triggered using optical and/or electrochemical inputs (Figure 29b). This multi‐addressable system is of great interest for the construction of molecular logic gates and molecular photometric memory devices.

**FIGURE 30:**
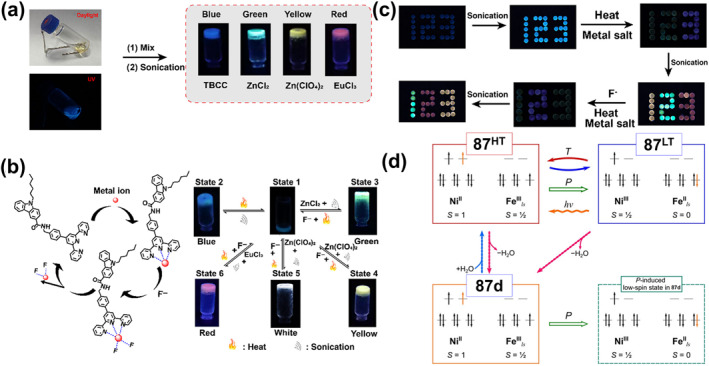
(a) Schematic preparation of organogels with different emission colors under UV light. (b) Schematic description of *in situ* control of emission colors through dynamic metal‐ligand (M‐L) coordination (left) and the interconversion among six states (non‐emissive, blue, green, yellow, red, and white) and associated photographs under UV irradiation (right). (c) The demonstration of reversible multicolor encoding and decoding. Reproduced with permission: copyright 2020, Chinese Chemical Society Publishing.^[213]^ (d) Multi‐responsivity of electron‐transfer in **87** to physical (solid lines) and chemical (dashed lines) stimuli of temperature (*T*; red‐heating, blue‐cooling), pressure (*P*), light (*hν*), dehydration (magenta) and rehydration (light blue). Reproduced with permission: copyright 2020, John Wiley and Sons.^[214]^

In addition to molecular logic gates, multi‐addressing systems based on molecular switches are also an effective way to regulate the chromophore luminescence conversion process. Despite preliminary successes in multicolor luminescence, most are based on mixed fluorophore systems and exhibit up to four emission states only. Controlling multicolor luminescence of a single chromophore is of great importance but remains a great challenge. To address this issue, in 2020, Ma et al. achieved the control of multicolor states and the creation of communication networks by exploiting the dynamic properties of sol‐gel transformations and M‐L coordination.^[213]^ They prepared a novel chromophore (N‐{4‐[(2,2′:6′,2″‐terpyridine)‐4′‐yl]benzyl}‐9‐hexyl‐9H‐carbazole‐3‐carboxamide, **86**) by doping acyl amides with carbazole moieties into the terpyridine, constructing a small‐molecule organogel with chelating motifs. This gel can be used in conjunction with different zinc salts to change the CT state within the molecule and thus the emission wavelength. Cooperation of the gel with europium ions produces characteristic luminescence, which further extends the emission tonal range (Figure 30a). The fluorescence‐emitting **86** can undergo sol‐gelation to the blue‐emitting state after heating, or it can exhibit green luminescence upon sonication stimulation with the addition of ZnCl_2_, and red emission can be achieved after replacing it with the metal ion EuCl_3_. After changing the counter ion, **86** can turn into yellow or white emission. Moreover, these processes are reversible under the stimulation of fluorine ions and heating (Figure 30b). Furthermore, the authors constructed the polymorphic emission colors to achieve dynamic multicolor encoding and decoding functions (Figure 30c). The multi‐addressable fluorescent molecules realized by such a simple and effective design strategy are promising for the design and development of photonic applications such as multi‐color displays, information encryption and decryption, and logic gates.

The above examples mainly focus on changes in redox states and optical properties, but to further extend the use of stimuli‐responsive molecular‐based materials multi‐stimuli‐responsive materials with magnetization and color changes are attracting widespread interest. CN‐bridged metal complex systems (i.e., M‐CN‐M′) are ideal candidates for the construction of multifunctional active molecular magnetic materials with multiple external stimuli due to the electron transfer phase transition (ETPT). However, in the most studied examples, especially the Co‐based compounds, magnetic bistability between the paramagnetic and diamagnetic state is achieved through thermal electron‐transfer‐coupled spin transition. In contrast, there are few examples in which the ETPT in CN‐bridged metal complexes is induced or modulated by applied pressure. In this context, Nowicka and colleagues reported a unique multi‐stable and multi‐reactive CN‐bridged coordination chain {NH_4_ [Ni(cyclam)]‐ [Fe(CN)_6_ ]‐5H_2_O}_n_ (**87**), where the coordination chains exist in both Ni^II^ ‐Fe^III^ ((**87** ^**HT**^) and Ni^III^‐Fe^II^ ((**87** ^**LT**^) valence states.^[214]^ These complexes exhibit unique multiple responses to various stimuli (including temperature, pressure, light, and humidity) under environmental conditions with significant changes in magnetization and color (Figure 30d). The two valence states **87** ^**HT**^ and **87** ^**LT**^ undergo conversion due to a hot ETPT. This phase transition exhibits a room temperature thermal hysteresis phenomenon that can be induced by irradiation and transferred to higher temperatures under small pressure treatments. In addition, it can be reversibly returned to the {NH_4_ [Ni^II^(cyclic amine)][Fe^III^(CN)_6_]}_n_ (**87d**) with Ni^II^‐Fe^III^ valence state by dehydration, while being transformed to the Ni^III^‐Fe^II^ state in response to pressure above 1.06 GPa. This responsive behavior toward numerous external stimuli in molecular magnetic materials also has great application in the field of multifunctional molecular switches, indicators and information converters or storage.

## 8 CONCLUSIONS AND PERSPECTIVE

Smart bistable coordination complexes are important fundamental units for the development of smart materials, and the research of this field is growing rapidly and is highly diverse. Herein, we reviewed the recent advances according to the different external stimuli that induce the switching process. Examples ranging from visualization of color transitions and emission color changes to crystalline transitions and magnetic changes are discussed for different applications with specific responses to environmental stimuli. In most of the examples, the complexes are linear in response to the external stimuli, that is, a stimulus elicits a specific response that produces a single stable state. These representative examples show that the response of the bistable materials can be rationally designed and controlled on demand in response to external stimuli. Of course, there are also smart materials that show multi‐addressability and multi‐responsiveness, which can further undergo chemical transitions in response to multiple stimuli and produce multiple steady states, facilitating the implementation of more complex systems.

In the past decades, we have witnessed various breakthroughs; however, there are still many challenges to overcome. First, the fatigue resistance and stability of these complex materials still have much room for development, which is ascribed to the energy consumption of the interconversion isomerization process. Secondly, the compatibility issues of bistable complexes with other materials during further device fabrication need to be further investigated. Third, due to the sensitivity of the complexes to the external environment and the diversity of testing conditions in different scenarios, there is a lack of specific evaluation criteria and measurement method for bistable complexes. Finally, the application scenarios of bistable smart complex materials need to be further specified and practical, such as the realization of human‐computer interactive through the signal transformation of bistable smart responsive materials or the detection, preservation and reset of signals in wearable devices by using bistable properties.

However, with the continuous development and innovation of material processes and the rapid improvement of various physical characterization techniques, many bistable smart complex materials with excellent performances have been reported and various more scientific and accurate characterization means have been implemented one after another. We hope that in the near future, through the efforts of researchers and industry workers together, as well as the communication and cooperation of different disciplines, the existing bottlenecks can be overcome, and more innovative designs can emerge to take advantage of hte enormous potential of these smart materials to promote their global commercialization for the better benefit of society.

## CONFLICT OF INTEREST STATEMENT

The authors declare no conflicts of interest.

## Data Availability

Data sharing is not applicable to this article as no new data were created or analyzed in this study.
